# Multi-environment evaluation and identification of Tartary buckwheat (*Fagopyrum tataricum* Gaertn.) genotypes for superior agronomic and nutritional potential in the North-Western Himalayas

**DOI:** 10.1038/s41598-025-15790-3

**Published:** 2025-08-22

**Authors:** Vivek Singh, Amit Rana, Shorya Kapoor, Rhitisha Sood, Shabnam Kumari, Shailja Sharma, Nimit Kumar, Indra Pratap Singh, Gopal Katna

**Affiliations:** 1 Department of Genetics and Plant Breeding, Chaudhary Sarwan Kumar Himachal Pradesh Krishi Vishvavidyalaya, Palampur, Himachal Pradesh 176062 India; 2 Department of Vegetable Science and Floriculture, Chaudhary Sarwan Kumar Himachal Pradesh Krishi Vishvavidyalaya, Palampur, Himachal Pradesh 176062 India; 3 Department of Agronomy, Chaudhary Sarwan Kumar Himachal Pradesh Krishi Vishvavidyalaya, Palampur, Himachal Pradesh 176062 India; 4 Mountain Agricultural Research and Extension Centre, Sangla, Himachal Pradesh 172106 India; 5 Department Animal Husbandry and Dairying, Shri Murli Manohar Town Post Graduate College, Ballia, Uttar Pradesh 277001 India

**Keywords:** Buckwheat, AMMI, GGE, WAAS, WAASBY, MTSI, Plant sciences, Environmental sciences

## Abstract

Tartary buckwheat (*Fagopyrum tataricum* (*L.*) Gaertn) is an important underutilized coarse cereal, grown for its excellent nutritional, health value and therapeutic effects. Despite its growing demand, there are limited studies that have focused on its genotypic variability and genotype-environmental interaction (GEI), particularly in the North-Western Himalayas. This study evaluated 24 Tartary buckwheat genotypes across five specific test environments (E1–E5) for 9 agronomic and 6 nutritional traits to estimate the effects of genotype (G), environment (E) and their interaction (GEI) using Additive Main Effects and Multiplicative Interaction (AMMI), Genotype and Genotype × Environment Interaction (GGE) biplot, Weighted Average of Absolute Scores (WAAS), Best Linear Unbiased Prediction (BLUP) and the Multi-Trait Stability Index. The results revealed significant GEI effects for all the evaluated traits. High heritability and genetic advance as a percentage of the mean for number of seeds per plant and seed yield per plant, suggested strong potential for genetic improvement. Based on mean performance, AMMI, WAAS, WAASBY and GGE analysis, genotypes G2, G13, G19, G1, G15 and G23 were found fairly stable alongside superior trait performance and nutritional content. Environmental analysis highlighted E2, E5 and E4 at Palampur (H.P.), as the most representative and discriminating environments. Multi-trait stability index analysis identified genotypes G2, G13, G1 and G19 as the most stable and ideal. These findings provide critical insights into the adaptability and performance of buckwheat genotypes under diverse agro-climatic conditions. Hence, these genotypes can serve as valuable resources for breeding programs aimed at developing high-yielding, nutritionally enhanced Tartary buckwheat varieties suitable for the North-Western Himalayan region.

## Introduction

Tartary buckwheat (*Fagopyrum tataricum* (*L.*) Gaertn), also known as bitter buckwheat, is a multi-purpose dicotyledonous crop belonging to the Polygonaceae family. It is a traditional short-season medicinal plant primarily cultivated for its highly nutritious seeds and is often classified as a pseudo-cereal^1^. This crop is predominantly grown in Asia (China, Bhutan, Nepal, India) as well as in America and Europe^2,3^. In terms of proximate composition, Tartary buckwheat contains approximately 70.2% starch, 10.5% protein, 2.8% fat, 2.6% crude fibre and 2.4% ash^2^. Additionally, it is rich in bioactive compounds, including carbohydrates, vitamins (B1, B2, B6, C, and E), polyphenols, anthraquinones, phenolic acids, flavonoids, carotenoids, minerals, lipids, etc.^2,4–6^. It also offers superior nutraceutical benefits compared to common buckwheat, with a higher total vitamin B content and substantially greater concentrations of antioxidants, particularly rutin^7^. These bioactive compounds contribute to numerous health benefits such as anti-bacterial, anti-genotoxicity, anti-cancer, anti-hypertension, anti-inflammatory, anti-diabetic, cholesterol-lowering and cognition-improving properties^8–10^. Furthermore, the seeds of this plant are naturally gluten-free, which makes it an excellent dietary choice for people with gluten intolerance or celiac disease^11^. It is widely used in various folk medicine applications and available in several food products such as noodles, vinegar, beverages, cakes and sprouts^1^.

Despite its multifaceted nutritional and agronomic potential, Tartary buckwheat remains an underutilized ‘orphan crop’ in agricultural systems. Several factors, including low and unstable seed yields, complex flowering biology, female sterility, sensitivity to environmental fluctuations, and susceptibility to pre-harvest sprouting, have hindered its broader adoption^12,13^. Developing genotypes with high adaptability and superior nutritional profiles is essential to overcome these limitations. Although Tartary buckwheat holds significant promise but only a few studies have explored its genotypic variability and genotype × environment interaction (GEI). Consequently, extensive evaluation of conserved germplasm for grain yield and other important traits becomes critical for its genetic improvement. However, the expression of these traits is often strongly influenced by GEI, leading to inconsistent performance across diverse environments. Environmental factors such as rainfall, temperature, soil chemistry, and humidity create a complex matrix that shapes genotype-environmental interaction GEI^14,15^. Therefore, understanding and quantifying these interactions is indispensable for breeding programs aiming to develop stable, high-yielding, and widely adaptable genotypes.

Multi-environment trials (METs) form the cornerstone of such evaluations, which help breeders to assess the performance of a genotype under varied conditions. Conventionally, analysis of variance (ANOVA) has been used to partition variability into components attributable to genotype, environment, and their interaction. However, its ability to capture the nuanced interactions between genotypes and environments is limited as it assumes a uniform variance–covariance structure across genotypes. To address these limitations, multivariate statistical tools such as additive main effects and multiplicative interaction (AMMI) models and genotype + genotype × environment (GGE) biplots have gained importance. These tools provide a more comprehensive analysis by incorporating the multiplicative nature of GEI, offering breeders a visual and statistical framework for selecting stable and high-performing genotypes^16,17^. A new technique, Weighted Average Absolute Scores (WAAS), has been introduced based on the AMMI model^18^. It integrates all interaction principal components (IPCs), thereby encompassing the full spectrum of GEI variance in genotype evaluation. This approach allows for a more thorough understanding of genotype stability and performance. Building upon WAAS, the WAASBY index (Weighted Average of Absolute Scores and Yield-Based Stability Index) incorporates both stability and yield, providing a balanced metric that aligns with the dual breeding objectives of high productivity and adaptability. Also, the utilization of linear mixed models (LMMs) has aided in the prediction of genotype performance in a certain environment, i.e. BLUP (Best Linear Unbiased Predictor). BLUP is used to estimate random effects while treating block and environment effects as fixed and genotype effects as random. This approach addresses the limitations of the AMMI model, offering a more accurate prediction of genotype performance across diverse environments^19,20^. Moreover, to identify stable and superior genotypes based on multiple traits, an advanced tool known as the Multi-Trait Stability Index (MTSI) has recently been applied in MET^21,22^. This method integrates both fixed and random effects models with factor analysis to evaluate genotypes across multiple traits. By calculating the distance from an ideal genotype, MTSI enables the simultaneous selection of stable genotypes with positive selection differentials for desirable traits and negative differentials for undesirable ones. Therefore, this integration of these tools enables breeders to screen genotypes that not only perform consistently across environments but also exhibit superior yield potential^23^. Hence, based on this methodological framework, the present investigation was conducted to evaluate GEI and identify stable, high-performing Tartary buckwheat genotypes and promote their large-scale cultivation in the North-Western Himalayan region.

## Results

### Genetic variation and pooled ANOVA across test environments

ANOVA of fifteen traits evaluated among 24 Tartary buckwheat genotypes in all five environments showed significant mean sum of squares demonstrating substantial differences among genotypes for all the traits evaluated (Supplementary Table 1). The homogeneity of variances over the years was tested using Bartlett’s test. The results of the test revealed that K-squared values for all the traits were non-significant (*p* > 0.05) over the years (Table 1). Pooled ANOVA indicated statistically significant differences among genotypes (G), environment (E) and genotype × environment interaction (GEI) for almost all 15 parameters studied, except for variations due to E for nutritional parameters viz*.,* P and Fe (Table 2). Among all the parameters assessed, P (39.76%) exhibited the most variance towards GEI to overall variability, followed by Mg (35.38%) and PC (35.28%). At the same time, genotypes shared maximum variation for most of the parameters, with the highest variation for NSPP (90.22%), followed by Fe (89.43%) and SYPP (87.81%). Further, a comparatively small contribution towards environmental variation was observed for most of the traits (< 20%) except Zn (31.88%), SPP (31.03%) and PH (28.33%).

**Table 1:** Bartlett’s test of homogeneity of variance.

Traits	d.f	Bartlett’s K-squared	*p* value	Traits	d.f	Bartlett’s K-squared	*p* value
		Agronomic				Nutritional	
DTF	4	4.24^ns^	0.37	Ca	4	2.78^ns^	0.60
DTM	4	0.46^ns^	0.98	P	4	2.31^ns^	0.68
LPP	4	9.36^ns^	0.06	Mg	4	2.32^ns^	0.68
PBP	4	0.34^ns^	0.99	Fe	4	3.10^ns^	0.54
PH	4	3.01^ns^	0.56	Zn	4	7.88^ns^	0.10
100-SW	4	1.45^ns^	0.84	PC	4	1.86^ns^	0.76
NSPP	4	2.30^ns^	0.68				
SPP	4	4.78^ns^	0.31				
SYPP	4	0.24^ns^	0.99				

ns: non-significant (*p* > 0.05).

DTF: Days to 50% flowering; DTM: Days to 80% maturity; LPP: Leaves per plant; PBP: Primary branches per plant; PH: Plant height; 100-SW: 100 seed weight; NSPP: Number of seed per plant; SPP: Straw yield per plant; SYPP: Seed yield per plant; Ca: Calcium; P: Phosphorus; Mg: Magnesium; Fe: Iron; Zn: Zinc; PC: Protein content.

**Table 2:** Pooled analysis of variance for fifteen agronomic and nutritional traits evaluated in 24 Tartary buckwheat genotypes over five environments (2021–23).

Source of variation	E (d.f.: 4)		G (d.f.: 23)		GEI (d.f.: 92)		R(E) (d.f.: 10)	Residuals (d.f.: 230)	CV (%)
Trait	MSS	% Var (G + E + GEI)	MSS	% Var (G + E + GEI)	MSS	% Var (G + E + GEI)	MSS	MSS	
DTF	181.82*	7.70	337.54*	82.14	10.44*	10.16	1.61	1.88	10.52
DTM	190.67*	7.21	372.86*	81.07	13.48*	11.72	4.96	5.19	6.48
LPP	199.29*	7.08	380.83*	77.76	18.56*	15.16	0.56	0.56	19.98
PBP	2.12*	10.53	2.31*	65.92	0.21*	23.55	0.02	0.01	11.57
PH	2,479.96*	28.33	1,020.52*	67.03	17.64*	4.64	5.82	4.97	16.66
100-SW	1.10*	17.28	0.80*	71.80	0.03*	10.92	0.00	0.00	12.25
NSPP	552.82*	0.77	11,319.91*	90.22	282.77*	9.01	6.99	10.24	23.24
SPP	23.12*	31.03	6.28*	48.49	0.66*	20.48	0.02	0.04	11.74
SYPP	5.52*	7.32	11.52*	87.81	0.16*	4.88	0.01	0.01	31.13
Ca	377.36*	14.88	221.30*	50.17	38.55*	34.95	3.09	2.05	9.98
P	72.83^ns^	0.17	4,426.52*	60.07	732.42*	39.76	33.31	98.72	6.41
Mg	472.49*	1.51	3,440.56*	63.11	482.13*	35.38	36.58	25.97	9.27
Fe	0.01^ns^	0.81	1.10*	89.43	0.12*	9.76	0.01	0.01	8.28
Zn	0.51*	31.88	1.03*	64.38	0.06*	3.75	0.01	0.01	10.19
PC	0.43*	0.14	34.80*	64.58	4.75*	35.28	0.07	0.11	14.44

ns: non-significant; *Significant at 5% level (*p* ≤ 0.05).

% Var: (G + E + GEI); DTF: Days to 50% flowering; DTM: Days to 80% maturity; LPP: Leaves per plant; PBP: Primary branches per plant; PH: Plant height; 100-SW: 100 seed weight; NSPP: Number of seed per plant; SPP: Straw yield per plant; SYPP: Seed yield per plant; Ca: Calcium; P: Phosphorus; Mg: Magnesium; Fe: Iron; Zn: Zinc; PC: Protein content; E: Environment; G: Genotype; GEI: Genotype environment interaction; R (E); Replications within environment.

### Trait performance, association analysis and genetic parameters

**Fig. 1:**
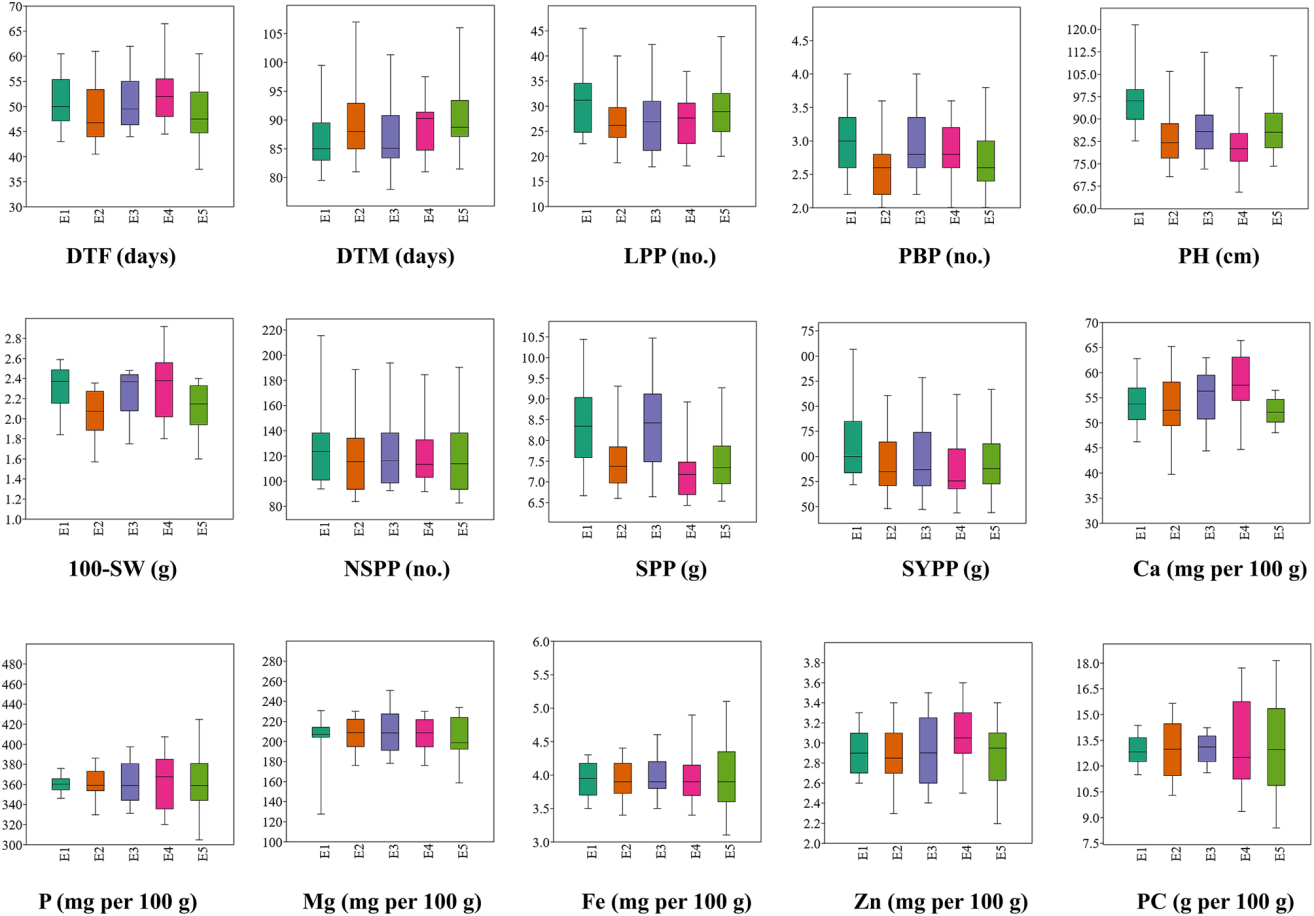
Box plot view of 15 agronomic and nutritional parameters evaluated in 24 Tartary buckwheat genotypes under five test environments during 2021–23 (DTF: Days to 50% flowering; DTM: Days to 80% maturity; LPP: Leaves per plant; PBP: Primary branches per plant; PH: Plant height; 100-SW: 100 seed weight; NSPP: Number of seed per plant; SPP: Straw yield per plant; SYPP: Seed yield per plant; Ca: Calcium; P: Phosphorus; Mg: Magnesium; Fe: Iron; Zn: Zinc; PC: Protein content).

The mean performance of Tartary buckwheat genotypes across five test environments over two years is presented in Supplementary Tables 2.1–2.5 (E1–E5) and visualized through boxplots in Fig. 1. Pooled means are summarized in Table 3, revealing substantial genotypic variation across all agronomic and nutritional traits. Genotypes G4, G18, G11, G3, and G23 flowered significantly earlier than the check G1 (Shimla B1), while only G5 matured earlier than the same check. For LPP and PH, none of the genotypes exceeded check performance. Genotypes G23, G5, G19, G11, G15, G13, and G7 recorded significantly higher PBP than the check G2 (Himpriya), while eleven genotypes were statistically at par with G2 for 100-SW. For NSPP, only G3 matched check G1, and no genotype surpassed check G1 for SPP and SYPP. Nutritional traits also showed notable variability; however, none of the genotypes exceeded check G1 in Ca and P, or check G2 in Mg, Fe, and Zn. For PC, G13 and G8 were significantly superior to check G2.

**Table 3:** Pooled mean performance observed in 24 Tartary buckwheat genotypes for 15 agronomic and nutritional parameters under five test environments during 2021–23.

G/ E	Pooled mean														
	Agro-morphological									Nutritional					
	DTF (days)	DTM (days)	LPP (no.)	PBP (no.)	PH (cm)	100-SW (g)	NSPP (no.)	SPP (g)	SYPP (g)	Ca (%/100 g)	P (%/100 g)	Mg (%/100 g)	Fe (%/100 g)	Zn (%/100 g)	PC (%/100 g)
G1	44.10^jk^	85.17^gh^	41.72^a^	2.28^mn^	110.30^a^	2.15^de^	194.58^a^	9.68^a^	5.25^a^	56.36^c-f^	371.84^b-f^	224.42^ab^	4.32^b^	3.28^a^	10.87^j^
G2	62.10^a^	102.26^a^	30.88^cd^	2.56^i-l^	100.52^b^	2.39^ab^	159.8^c^	8.38^bc^	3.69^cd^	54.72^e-h^	341.32^i-k^	225.76^ab^	4.64^a^	3.34^a^	13.26^ef^
G3	46.20^h-j^	84.66^gh^	26.62^f.^	2.36^l-n^	87.05^g-k^	2.29^c^	184.68^b^	8.00^de^	4.12^b^	49.9^kl^	357.72^e-i^	192.38^hi^	3.80^d-i^	3.04^cd^	10.89^j^
G4	42.00^k*^	83.47^gh^	31.32^cd^	2.84^fg^	90.49^c-g^	1.85^h^	122.69^f^	7.85^d-f^	2.51^gh^	59.52^ab^	354.12^f-j^	212.64^cd^	4.06^c^	2.82^f.-i^	13.77^de^
G5	48.80^e-g^	80.71^h^	30.90^cd^	3.48^b^	80.06^n-p^	2.23^cd^	112.39^g^	7.83^d-f^	2.19^jk^	55.32^d-g^*	369.02^b-f^*	204.68^d-f^	3.68^i^	2.80^g-k^	14.82^b^*
G6	54.20b	90.54^c-e^	27.30^f.^	2.96^ef^*	87.89^f.-j^	1.99^fg^	98.65^jk^	7.40^g-i^	1.64^l^	60.44^a^*	392.62^a^*	183.28^j^	3.70^hi^	2.40^n^	13.30^ef^*
G7	46.70^f.-i^*	86.29^fg^*	24.08^gh^	3.00^ef^*	82.52^l-n^	1.82^hi^	110.25^gh^	6.97^j^	2.22^ij^	50.48^kl^	337.22^jk^	228.26^a^*	3.78^e-i^	3.28^a^*	11.08^j^
G8	55.40^b^	86.13^fg^*	23.06^hi^	2.16^n^	87.57^g-k^*	2.23^cd^*	136.58^de^*	8.53^bc^*	2.69^f.^	57.46^b-d^*	381.12^a-d^*	228.62^a^*	3.90^c-g^	2.68^i-m^	15.54^a^*
G9	50.10^de^	84.59^gh^*	22.34^i^	2.48^j-m^	77.81^pq^	2.29^c^	115.35^g^	8.00^de^*	2.35^h-j^	55.70^d-g^*	329.02^ k^	195.78^f.-i^	3.74^f.-i^	2.96^d-f^*	14.19^cd^*
G10	53.20^bc^	87.00^e-g^*	26.94^f.^	2.48^j-m^	80.25^n-p^	2.09^e^	134.79^de^*	7.02^ij^	3.57^d^*	46.28^m^	348.62^g-k^	201.6^e-h^	3.82^d-i^	2.96^d-f^*	11.52^ij^
G11	46.30^g-j^*	84.89^gh^*	31.18^cd^*	3.24^cd^*	91.89^c-f^*	2.44^a^*	140.12^d^*	7.75^e-g^	3.58^d^*	58.50^a-c^*	372.82^b-f^*	197.66^f.-h^	3.94^c-e^	2.82^f.-j^	13.54^e^*
G12	54.60^b^	89.58^d-f^	28.82^e^*	2.72^g-i^	89.25^e-h^*	2.49^a^*	115.25^g^	7.90^d-f^*	2.70^f.^	51.10^j-l^	365.82^c-g^*	194.90^g-i^	3.90^c-g^	2.76^h-l^	11.37^j^
G13	54.70^b^	92.02^cd^	30.30^cd^*	3.08^de^*	94.45^c^*	2.08^ef^	99.22^jk^	7.35^h-j^	3.81^c^*	57.60^b-d^*	371.82^b-f^*	226.28^ab^*	4.26^b^*	3.36^a^*	15.88^a^*
G14	53.20^bc^	92.56^cd^	30.74^cd^*	2.52^i-l^	85.96^h-l^	1.72^i^	103.91^ij^	7.40^g-i^	1.58^l^	60.66^a^*	363.62^d-h^*	211.48^cd^*	3.72^g-i^	2.94^d-g^*	14.49^bc^*
G15	53.30^bc^	92.4^cd^	33.60^b^*	3.24^cd^*	84.54^j-m^	2.46^a^*	98.54^jk^	8.19^cd^*	2.04^k^	49.54^l^	359.42^e-i^	187.88^ij^	3.42^j^	2.60^m^	12.82^fg^
G16	49.10^ef^*	91.05^c-e^	31.62^c^*	2.68^g-j^	92.24^c-e^*	2.32^bc^*	105.74^hi^	7.83^d-f^*	2.66^fg^	55.06^d-g^*	349.72^g-j^	203.68^d-g^	4.04^c^*	2.88^e-h^	12.62^gh^
G17	47.10^f.-i^*	85.84^fg^*	21.10^j^	2.60^h-k^	84.98^i-m^	2.43^a^*	103.21^ij^	6.95^j^	2.82^f.^	51.62^i-l^	347.02^h-k^	198.48^f.-h^	3.98^cd^*	2.82^f.-j^	11.50^ij^
G18	44.10^jk^*	90.63^c-e^	23.20^hi^	2.84^fg^*	74.37^q^	2.06^ef^	130.93^e^*	7.84^d-f^*	3.36^e^*	52.22^h-k^	378.42^a-d^*	218.98^bc^*	4.34^b^*	2.66^lm^	12.48^gh^
G19	55.40^b^	97.25^b^	26.80^f.^	3.40^bc^*	83.7^k-n^	2.43^a^*	124.76^f.*^	7.13^ij^	3.29^e^*	59.22^ab^*	385.32^ab^*	210.68^c-e^*	4.36^b^*	3.30^a^*	12.07^hi^
G20	47.60^e-i^	87.38^e-g^	19.64^j^	2.56^i-l^	78.03^o-q^	2.32^bc^	135.13^de^	7.31^h-j^	3.54^d^	53.18^g-j^	346.72^h-k^	198.42^f.-h^	3.84^d-i^	2.82^f.-i^	14.86^b^
G21	47.6^e-i^	84.08^gh^	25.18^g^	2.88^e-g^	81.83^m-o^	1.91^gh^	135.67^de^	7.57^fh^	2.38^hi^	53.18^g-j^	340.82^ik^	204.58^d-f^	3.88^c-h^	3.22^ab^	12.55^gh^
G22	48.00^e-h^	85.8^fg^	30.20^d^	2.80^f.-h^	88.91^e-i^	2.06^ef^	91.45^l^	8.37^bc^	2.04^k^	50.82^j-l^	346.32^h-k^	231.88^a^	3.80^d-i^	3.10^bc^	11.53^ij^
G23	45.20^ij^	84.09^gh^	34.34^b^	3.68^a^	94.44^cd^	2.47^a^	90.94^l^	8.71^b^	2.27^ij^	53.76^f-i^	373.32^b-e^	194.58^g-i^	3.92^c-f^	2.98^c-e^	14.77^b^
G24	51.70^cd^	94.34^bc^	22.62^i^	2.40^k-m^	74.00^q^	2.45^a^	93.65^kl^	7.04^ij^	2.2^jk^	57.02^b-e^	384.02^a-c^	181.48^j^	3.88^c-h^	2.64^lm^	12.23^gh^
E1	50.75^2^	86.45^3^	30.55^1^	96.21^1^	2.95^1^	2.32^1^	126.83^1^	8.38^1^	3.32^1^	54.07^3^	359.74^1^	206.17^2^	3.94^2^	2.90^2^	12.92^3^
E2	48.25^3^	89.38^12^	26.79^3^	83.33^3^	2.53^4^	2.05^5^	120.79^3^	7.48^2^	2.76^3^	53.52^3^	360.65^1^	207.83^12^	3.94^2^	2.88^2^	12.93^23^
E3	50.44^2^	87.01^3^	27.05^3^	86.65^2^	2.93^1^	2.27^3^	123.48^2^	8.42^1^	2.87^2^	54.84^2^	361.26^1^	208.98^1^	3.97^1^	2.92^2^	12.99^1–3^
E4	52.13^1^	89.15^2^	27.00^3^	80.71^4^	2.85^2^	2.30^2^	120.17^3^	7.21^3^	2.57^4^	58.27^1^	361.78^1^	207.63^12^	3.94^2^	3.08^1^	13.04^12^
E5	48.58^3^	90.25^1^	29.14^2^	87.07^2^	2.75^3^	2.10^4^	120.88^3^	7.47^2^	2.75^3^	52.15^4^	362.36^1^	202.38^3^	3.95^12^	2.90^2^	13.10^1^
G.M	50.03	88.45	28.10	2.80	86.79	2.21	122.43	7.79	2.85	54.57	361.16	206.60	3.95	2.94	13.00
C.D	2.304	2.701	3.129	3.05	0.33	0.126	12.211	0.592	0.287	1.583	1.583	15.951	0.249	0.17	1.583
S.E. (m)	0.819	0.96	1.112	1.084	0.166	0.045	4.34	0.21	0.102	0.563	0.563	5.67	0.088	0.06	0.563
C.V	3.66	2.427	8.848	2.793	9.375	4.557	7.927	6.036	7.999	9.683	9.683	6.137	5.006	4.596	9.683

G: Genotypes; E: Environments; DTF: Days to 50% flowering; DTM: Days to 80% maturity; LPP: Leaves per plant; PBP: Primary branches per plant; PH: Plant height; 100-SW: 100 seed weight; NSPP: Number of seed per plant; SPP: Straw yield per plant; SYPP: Seed yield per plant; Ca: Calcium; P: Phosphorus; Mg: Magnesium; Fe: Iron; Zn: Zinc; PC: Protein content.

**Fig. 2:**
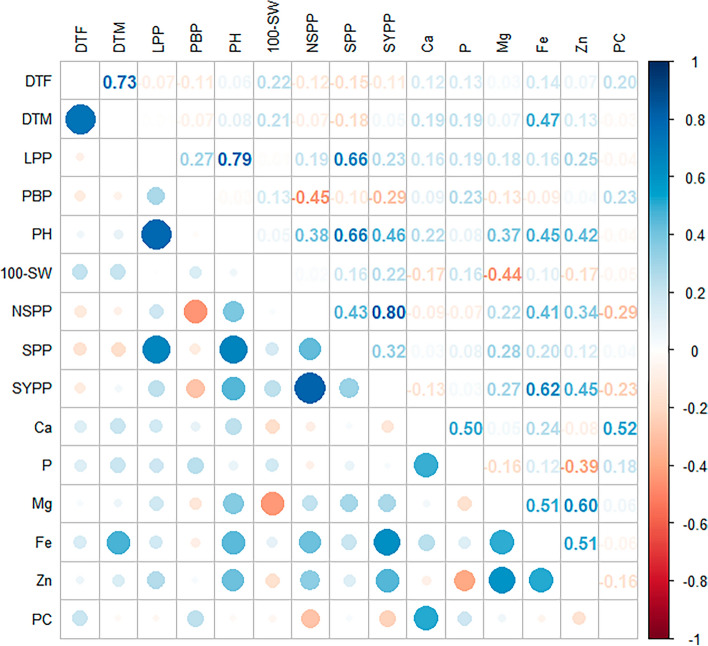
Trait associations among 15 agronomic and nutritional parameters evaluated in 24 Tartary buckwheat genotypes under five test environments.

Correlation studies among agronomical traits revealed that SYPP had a significant and positive correlation with NSPP and Fe (Fig. 2). Also, LPP had a significant and positive correlation with PH and SPP, whereas DTF was found to have a significant positive association with DTM. Additionally, among nutritional traits, Ca showed a significant and positive correlation with PC and P. While Mg had a significant positive correlation with Fe and Zn. Also, a significant positive association with each other was found between Fe and Zn. Various variability parameters were assessed across different environments and pooled data (Table 4). High phenotypic coefficient of variation (PCV) values (> 20%) were recorded for LPP, PBP, N and SYPP. Whereas traits such as DTF, PH, 100-SW, SYPP, Ca, P, Mg, Fe, and Zn exhibited moderate PCV values (10–20%). Similarly, high genotypic coefficient of variation (GCV) values (> 20%) were observed only for NSPP and SYPP, while moderate GCV values (10–20%) were noted for LPP, PBP, 100-SW and PC. Additionally, high broad-sense heritability (h^2^_bs_) values (> 60%) were observed for PH, NSPP and SYPP. While traits such as DTF, DTM, LPP, 100-SW and Zn showed moderate heritability (30–60%). High genetic advance as a percentage of the mean (GAM) (> 20%) was observed for LPP, NSPP and SYPP. The traits DTF, PBP, PH, 100-SW, Zn and PC showed moderate GAM values (10–20%).

**Table 4:** Variability parameters estimated in 24 Tartary buckwheat genotypes for 15 agronomic and nutritional parameters under five test environments during 2021–23 (E1-E5).

	E	DTF	DTM	LPP	PBP	PH	100-SW	NSPP	SPP	SYPP	Ca	P	Mg	Fe	Zn	PC
GCV (%)	E1	8.90	5.71	18.9	13.91	8.49	9.51	23.90	10.93	30.08	7.45	1.73	9.21	5.86	7.29	5.88
	E2	11.10	6.61	18.56	15.30	10.22	11.18	24.66	8.09	28.39	10.90	3.72	7.74	6.86	9.49	12.58
	E3	9.43	6.00	22.30	16.37	10.04	9.24	22.51	11.16	32.61	10.86	5.66	9.80	6.52	11.20	5.85
	E4	9.40	4.71	19.12	14.50	9.91	13.97	20.70	8.24	31.05	9.28	6.99	7.70	8.24	8.93	20.06
	E5	10.80	6.01	18.36	17.69	10.04	10.91	25.22	8.45	29.20	5.01	8.77	10.50	11.54	11.06	20.35
	Pooled	9.35	5.47	17.41	12.88	9.4	10.22	22.13	7.73	29.44	6.36	4.29	6.77	6.45	8.64	10.87
PCV (%)	E1	9.23	6.30	19.11	14.39	8.86	9.78	24.03	11.13	30.22	7.95	3.23	9.59	6.28	7.73	6.43
	E2	11.37	7.12	18.73	15.93	10.48	11.48	24.81	8.48	28.57	11.17	4.86	8.05	7.22	9.97	12.78
	E3	9.90	6.50	22.45	16.76	10.40	9.52	22.64	11.52	32.75	11.20	6.23	10.06	7.02	11.58	6.43
	E4	9.79	5.38	19.25	15.04	10.25	14.17	20.84	8.71	31.25	9.65	7.44	8.14	8.69	9.19	20.24
	E5	11.21	6.49	18.59	18.14	10.37	11.29	25.39	8.87	29.38	5.58	9.19	10.80	11.82	11.28	20.50
	Pooled	12.53	7.85	26.35	24.08	11.38	14.5	28.39	15.48	34.28	16.08	10.70	15.37	13	13.51	24.27
h^2^_bs_ (%)	E1	93.12	82.18	97.81	93.37	91.81	94.6	98.91	96.31	99.10	87.90	28.74	92.28	86.99	88.92	83.54
	E2	95.32	86.13	98.22	92.24	95.07	94.77	98.77	90.91	98.74	95.25	58.69	92.35	90.34	90.67	96.90
	E3	90.79	85.11	98.66	95.40	93.19	94.23	98.84	93.77	99.11	93.95	82.59	94.98	86.39	93.40	82.91
	E4	92.18	76.49	98.67	92.92	93.54	97.17	98.67	89.42	98.72	92.46	88.28	89.57	89.82	94.39	98.26
	E5	92.74	85.85	97.47	95.11	93.69	93.47	98.67	90.80	98.76	80.54	91.19	94.51	95.34	96.24	98.49
	Pooled	55.73	48.61	43.65	28.58	68.19	49.64	60.77	24.94	73.73	15.66	16.05	19.4	24.59	40.9	20.06
GAM (%)	E1	17.69	10.66	38.51	27.68	16.76	19.06	48.97	22.09	61.69	14.39	1.91	18.23	11.25	14.16	11.07
	E2	22.33	12.64	37.89	30.27	20.53	22.41	50.48	15.89	58.12	21.91	5.87	15.31	13.44	18.62	25.50
	E3	18.52	11.40	45.63	32.93	19.97	18.48	46.10	22.25	66.87	21.68	10.60	19.68	12.49	22.29	10.98
	E4	18.59	8.48	39.12	28.79	19.74	28.37	42.35	16.05	63.55	18.38	13.53	15.01	16.08	17.88	40.96
	E5	21.42	11.47	37.33	35.55	20.02	21.74	51.61	16.59	59.77	9.26	17.26	21.03	23.21	22.35	41.60
	Pooled	14.38	7.86	23.70	14.18	15.99	14.83	35.53	7.95	52.07	5.19	3.54	6.14	6.59	11.38	10.03

DTF: Days to 50% flowering; DTM: Days to 80% maturity; LPP: Leaves per plant; PBP: Primary branches per plant; PH: Plant height; 100-SW: 100 seed weight; NSPP: Number of seed per plant; SPP: Straw yield per plant; SYPP: Seed yield per plant; Ca: Calcium; P: Phosphorus; Mg: Magnesium; Fe: Iron; Zn: Zinc; PC: Protein content; h^2^_bs_: Heritability (broad sense); GCV: Genotypic Co-efficient of Variation; PCV: Phenotypic Co-efficient of Variation (%); GAM: Genetic Advance as per cent Mean (%).

### AMMI ANOVA

AMMI ANOVA analysis for all the agronomic and nutritional parameters revealed significant differences among all the Tartary buckwheat genotypes, five test environments and their interaction effect, except for P and Fe under environmental effect (Table 5). By partitioning the total variation, it was observed that genotypic effects explained the majority of the variation for all the evaluated traits. Among agronomic traits, the highest genotypic effect was observed in NSPP (89.475 of the total S.S.) followed by SYPP (87.19%) and DTF (78.71%) whereas among nutritional traits, Zn (73.47%), Fe (65.8%) and PC (63.26%) had the highest genotypic effect. However, the environmental effects accounted for a small proportion of the total variation. The highest environmental effect among agronomic traits was SYPP (30.02%), PH (27.40%) and 100-SW (16.87%), while Ca (14.19%) and Zn (6.31%) among nutritional traits. The variation due to genotype × environment interaction among agronomic traits revealed the highest interaction contribution by PBP (22.65%) followed by SPP (19.80%) and LPP (14.98%). Similarly, among nutritional traits, P (35.00%), PC (34.55%), and Ca (33.29%) had the highest contribution to genotype × environment interaction. Upon further partitioning of GEI into interaction principal components (IPCAs), it was found that by IPCA1 and IPCA2 in combination explained ≥ 80% of the GEI (DTF: 89.60%; DTM: 94.20%; LPP: 94.30%; PBP: 82.30%; PH: 95.00%; 100-SW: 92.90%; NSPP: 94.00%; SPP: 88.30%; SYPP: 87.20%; Ca: 89.60%; P: 88.10%; Mg: 84.40%; Fe: 93.50%; Zn: 80.80%; PC: 90.20%). Also, the subsequent PCs (PC3 and PC4) captured progressively smaller contributions.

**Table 5:** Analysis of variance based on AMMI (Additive Main Effects and Multiplicative Interaction) model for 15 agronomic and nutritional parameters under five test environments during 2021–23 (E1-E5).

Source	d.f	DTF		DTM		LPP		PBP		PH	
		MSS	% (SS)	MSS	% (SS)	MSS	% (SS)	MSS	% (SS)	MSS	% (SS)
Environment (E)	4	186.03*	7.48	191.52*	6.50	199.29*	6.99	2.12*	10.13	2480.43*	27.40
Rep (E)	10	1.69	0.17	4.41	0.37	0.56	0.05	0.02	0.24	5.81	0.16
Genotype (G)	23	340.26*	78.71	370.36*	72.23	380.83*	76.84	2.31*	63.40	1020.55*	64.81
G × E	92	10.06*	9.31	13.82*	10.78	18.56*	14.98	0.21*	22.65	17.63*	4.48
Residuals	230	1.87		5.19		0.56		0.01		4.96	
Total	451	24.10		28.97		29.06		0.23		83.90	
		Var (%)	C. Var (%)	Var (%)	C. Var (%)	Var (%)	C. Var (%)	Var (%)	C. Var (%)	Var (%)	C. Var (%)
PC1	26	24.30 (68.30)	68.30	37.27 (76.20)	76.20	44.07 (67.10)	67.10	0.46 (62.60)	62.60	44.81 (71.80)	71.80
PC2	24	8.51 (22.10)	90.30	9.54 (18.00)	94.20	19.39 (27.20)	94.30	0.16 (19.70)	82.30	15.63 (23.10)	95.00
PC3	22	3.22 (7.70)	98.00	2.55 (4.40)	98.60	2.52 (3.20)	97.60	0.10 (11.30)	93.50	3.11 (4.20)	99.20
PC4	20	0.93 (2.00)	100.00	0.87 (1.40)	100.00	2.06 (2.40)	100.00	0.06 (6.50)	100.00	0.67 (0.80)	100.00

Source	d.f	100-SW		NSPP		SPP		SYPP		Ca	
		MSS	% (SS)	MSS	% (SS)	MSS	% (SS)	MSS	% (SS)	MSS	% (SS)
Environment (E)	4	1.11*	16.87	553.35*	0.76	23.13*	30.02	5.52*	7.26	377.71*	14.19
Rep (E)	10	0.00	0.10	6.97	0.02	0.02	0.06	0.01	0.03	3.06	0.29
Genotype (G)	23	0.80*	69.77	11,320.02*	89.47	6.28*	46.91	11.52*	87.19	221.35*	47.81
G × E	92	0.03*	10.64	282.74*	8.94	0.66*	19.80	0.16*	4.84	38.53*	33.29
Residuals	230	0.00		10.24		0.04		0.01		2.05	
Total	451	0.06		702.94		0.82		0.71		31.47	
		Var (%)	C. Var (%)	Var (%)	C. Var (%)	Var (%)	C. Var (%)	Var (%)	C. Var (%)	Var (%)	C. Var (%)
PC1	26	0.07 (66.40)	66.40	733.26 (73.30)	73.30	1.75 (74.80)	74.80	0.41 (72.70)	72.70	74.86 (54.90)	54.90
PC2	24	0.03 (26.50)	92.90	224.63 (20.70)	94.00	0.34 (13.50)	88.30	0.09 (14.50)	87.20	51.27 (34.70)	89.60
PC3	22	0.01 (6.50)	99.40	52.29 (4.40)	98.40	0.28 (10.10)	98.30	0.06 (8.90)	96.00	14.57 (9.00)	98.70
PC4	20	0.00 (0.00)	100.00	20.29 (1.60)	100.00	0.05 (1.70)	100.00	0.03 (4.00)	100.00	2.36 (1.30)	100.00

Source	d.f	P		Mg		Fe		Zn		PC	
		MSS	% (SS)	MSS	% (SS)	MSS	% (SS)	MSS	% (SS)	MSS	% (SS)
Environment (E)	4	73.89	0.15	471.88*	1.43	0.01	0.11	0.51*	6.31	0.45*	0.14
Rep (E)	10	33.81	0.18	36.85	0.28	0.01	0.14	0.01*	0.37	0.07*	0.06
Genotype (G)	23	4427.02*	52.89	3440.53*	60.08	1.10*	65.84	1.03*	73.47	34.80*	63.26
G × E	92	732.36*	35.00	482.16*	33.68	0.12*	28.11	0.05*	15.65	4.75*	34.55
Residuals	230	98.69		25.96		0.01		0.01		0.11	
Total	451	576.29		390.41		0.11		0.08		3.77	
		Var (%)	C. Var (%)	Var (%)	C. Var (%)	Var (%)	C. Var (%)	Var (%)	C. Var (%)	Var (%)	C. Var (%)
PC1	26	1824.16 (70.40)	70.40	1077.95 (63.20)	63.20	0.32 (76.20)	76.20	0.11 (55.50)	55.50	12.84 (76.40)	76.40
PC2	24	496.84 (17.70)	88.10	392.81 (21.30)	84.40	0.08 (17.30)	93.50	0.05 (25.30)	80.80	2.51 (13.80)	90.20
PC3	22	233.82 (7.60)	95.70	313.85 (15.60)	100.00	0.03 (6.50)	100.00	0.04 (19.20)	100.00	1.84 (9.20)	99.40
PC4	20	144.02 (4.30)	100.00	0.00 (0.00)	100.00	0.00 (0.00)	100.00	0.00 (0.00)	100.00	0.13 (0.60)	100.00

Significant at 5% level (*p* ≤ 0.05)

DTF: Days to 50% flowering; DTM: Days to 80% maturity; LPP: Leaves per plant; PBP: Primary branches per plant; PH: Plant height; 100-SW: 100 seed weight; NSPP: Number of seed per plant; SPP: Straw yield per plant; SYPP: Seed yield per plant; Ca: Calcium; P: Phosphorus; Mg: Magnesium; Fe: Iron; Zn: Zinc; PC: Protein content; SS: Sum of squares; Var: Variance; C. Var: Cumulative Variance; PC: Principal Component

### Mean vs WAAS biplot

**Fig. 3:**
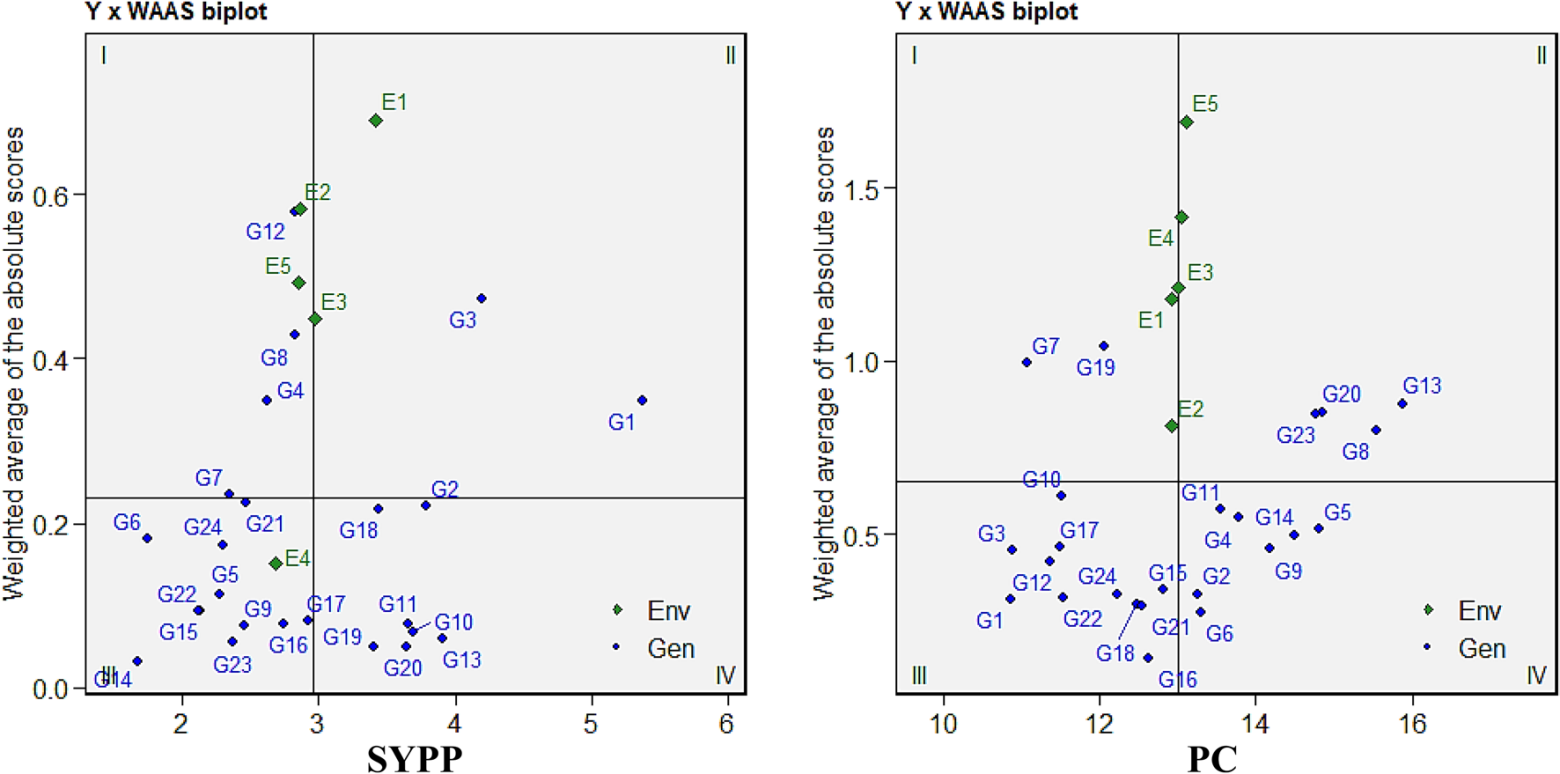
Mean × WAAS (Weighted Average of Absolute Scores) biplot of SYPP and PC among 24 Tartary buckwheat genotypes under five test environments.

The mean vs. WAAS biplot (Fig. 3; Supplementary Fig. 1) classifies genotypes and environments across four quadrants based on their mean performance (x-axis) and stability (y-axis, as indicated by WAAS values). Detailed classification of the genotypes across four quadrants is mentioned in Supplementary table 3. The first quadrant of the biplot has genotypes with below-average trait performance and high WAAS values. For example, genotypes such as G16 (DTF), G4, G10, G23 (DTM), G3, G8, G18 (LPP), and G19 (PH) fell in this group. Among nutritional traits, unstable and low-performing genotypes included G10 (Ca), G2, G9 (P), G20, G12 (Mg), and G6, G15 (Zn, PC). In quadrant II, genotypes with high mean trait performance but poor stability were located. These included G10, G15 (DTF), G2, G19 (DTM), G11, G23 (LPP), and G6, G12, G23 (PH). For nutritional traits, high-performing but unstable genotypes were G6, G24 (P), G2 (Fe), and G23, G13 (PC). No genotypes fell into this category for Zn. While quadrant III featured genotypes exhibited below-average trait performance and high stability (low WAAS values). Stable but low-performing genotypes included G1, G21 (DTF), G7, G11 (DTM), G24, G10 (LPP), and G9, G17 (PH). Stable but nutritionally underperforming lines included G3, G21 (Ca), G4, G14 (P), G5, G17 (Mg), and G7, G9 (Fe, Zn, PC). The last quadrant IV contained the most desirable genotypes, i.e. those with above-average performance and high stability. Noteworthy examples include G9, G13, G2 for DTF; G6, G14, G13 for DTM; G13, G4, G5, G15 for LPP; and G4, G7, G19 for PBP. Highly stable and productive genotypes across multiple agronomic traits also included G1, G2, G3, G11, G19 for PH, 100-SW, NSPP, and SYPP. For nutritional quality, genotypes such as G1, G5, G8, G14 (Ca, P), G2, G4, G14, G19 (Mg, Fe, Zn), and G4, G2, G6, G11, G14 (PC) stood out for their consistent and high-quality performance.

Overall, genotypes like G13, G14, and G19, along with both checks G2 and G1, repeatedly appeared in Quadrant IV for both agronomic and nutritional traits, indicating their potential for stable performance across diverse environments.

### WAASBY

**Fig. 4:**
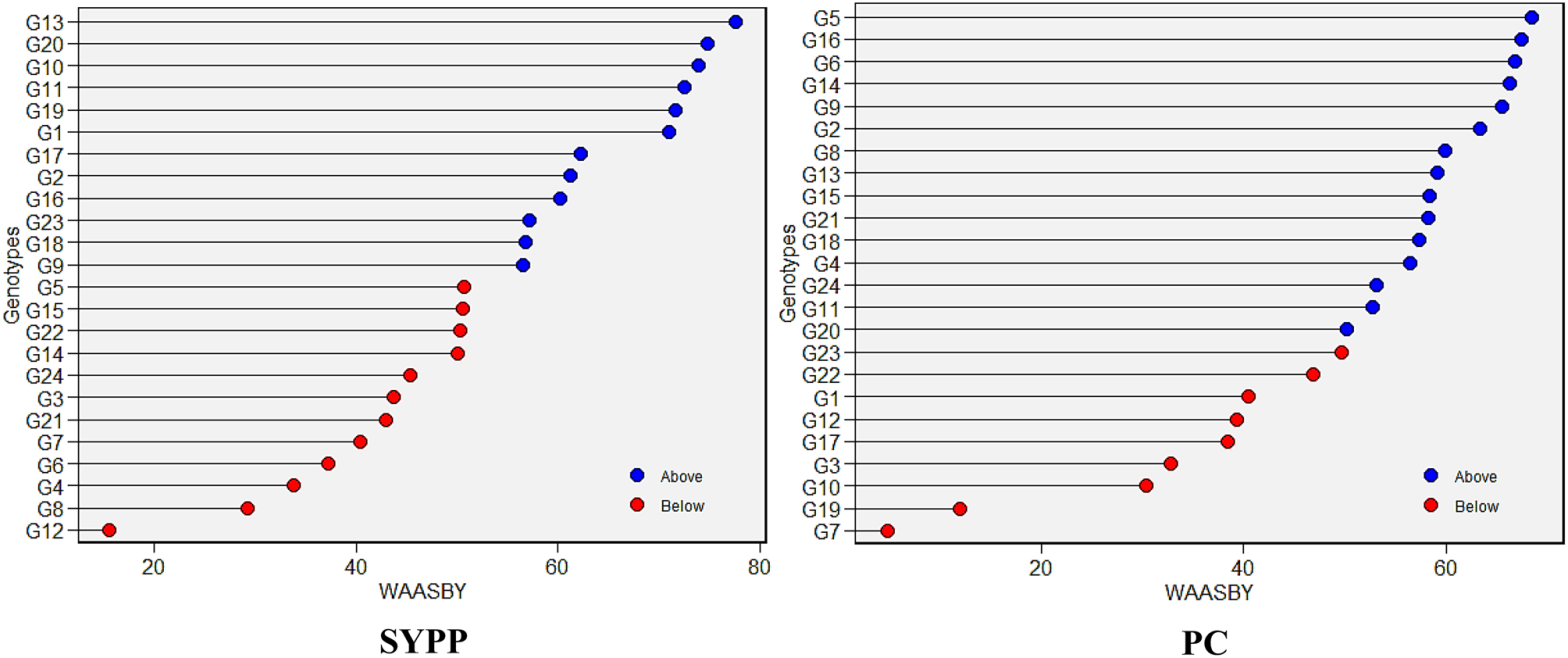
WAASBY (Weighted Average of Absolute Scores and Yield-Based Stability Index) based genotype ranking for SYPP and PC among 24 Tartary buckwheat genotypes under five test environments.

The WAASBY Index is a selection metric that integrates both the mean performance and stability of genotypes. Hence, the genotypes were likewise categorised based on the WAASBY index (Fig. 4 and Supplementary Fig. 2). The genotypes having values above mean WAASBY index are highlighted in blue circles, whereas the genotypes with lower values of the mean WAASBY index are marked in red colour. In this study, several genotypes consistently demonstrated high WAASBY indices across traits. Among the evaluated genotypes, check G2 (Shimla B1) ranked highest for traits DTF, PH, and Zn. Likewise, G19 emerged as a strong performer for DTF, PBP, and Ca. Check G1 (Himpriya) and G15 showed a desirable balance of productivity and stability in traits like LPP, NSPP, and PC. Additionally, G13, G14, and G8 demonstrated superior stability in nutritional quality traits such as SYPP, Ca, and P, respectively. Conversely, genotypes with the lowest WAASBY scores, including G10, G4, G8, G3, and G6, were unstable and exhibited suboptimal trait performance across multiple environments. These genotypes may require further evaluation before consideration in breeding pipelines.

### GGE biplot

#### Mean vs. stability

**Fig. 5:**
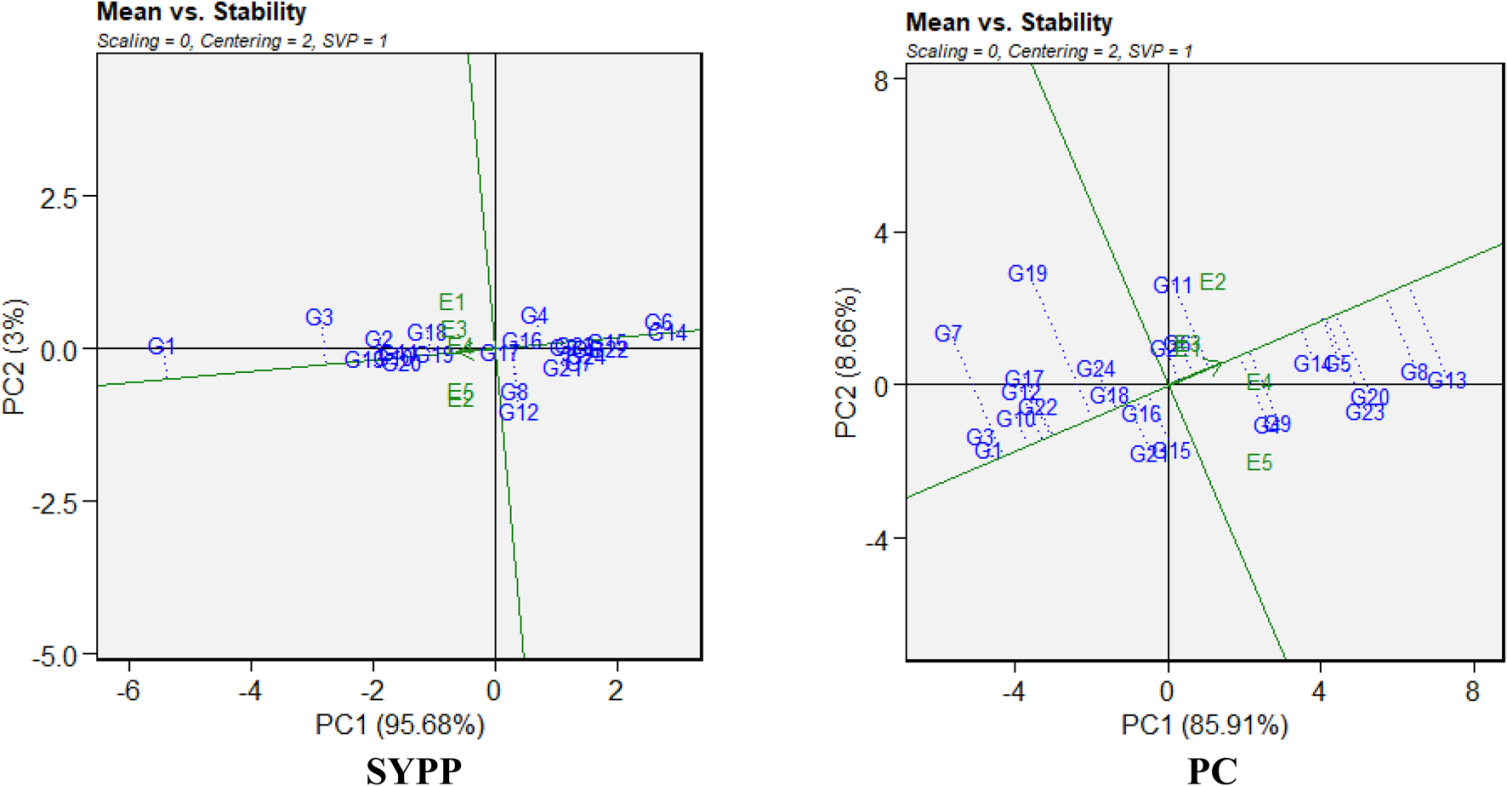
GGE (Genotype and Genotype × Environment Interaction) biplot of mean vs stability pattern for SYPP and PC among 24 Tartary buckwheat genotypes under five test environments.

The ‘mean vs. stability’ biplots (Fig. 5; Supplementary Fig. 3) explained a high proportion of total variation on PC1 and PC2 axes for all traits, ranging from 87.86% (Mg) to 98.63% (SYPP), indicating the reliability of biplot-based interpretations. Several genotypes consistently exhibited above-average trait values across multiple parameters. Check G1 was superior for eight traits, including LPP, PH, NSPP, SPP, SYPP, Mg, Fe and Zn, while check G2 also showed above-average performance for eight traits, viz*.,* PH, NSPP, SPP, SYPP, Mg, Fe, Zn and P. Likewise, G23 excelled in DTM, LPP, PH, PBP, 100-SW, SPP, SYPP and PC. G13 was notable for PC, Mg, Fe, Zn and PH, whereas G19 consistently performed well for Ca, PBP, P, Fe and Zn. Additional multi-trait performers included G8 (SPP, P, Mg, PC), G15 (LPP, PBP, 100-SW, SPP), G5 (DTM, PBP, PC), G4 (DTF, DTM, Ca), and G6 (Ca, P, PC). These genotypes can be considered for selection due to their strong performance across a wide range of agronomic and quality traits. Stability patterns across environments further differentiated genotypes. Those with minimal projection from the abscissa were found to be more stable, while genotypes with larger projections exhibited greater genotype × environment interaction, indicating variability in performance and lower stability.

#### Discriminativeness vs Representativeness

**Fig. 6:**
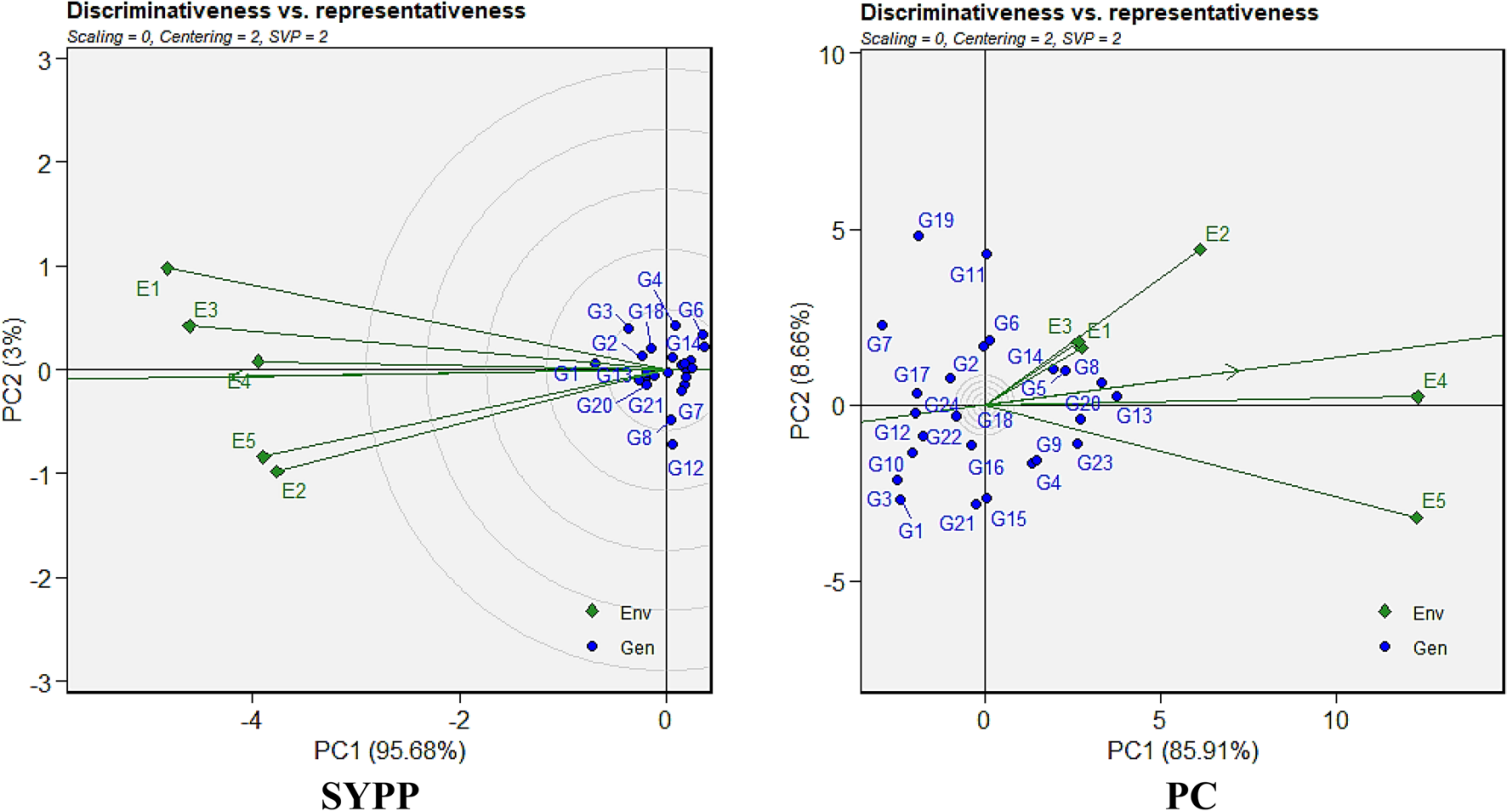
GGE (Genotype and Genotype × Environment Interaction) biplot of discriminativeness vs representativeness pattern for SYPP and PC among 24 Tartary buckwheat genotypes under five test environments.

Based on the genotype ranking patterns depicted in Fig. 6 and Supplementary Fig. 4, genotypes with optimal trait performance across individual environments were identified. Genotypes forming acute angles with all, or most, environments indicated superior average performance across multiple traits. The most ideal test environments are those that display the longest vector (high discriminative ability) and align closely along the average environment coordination (AEC) abscissa line, representing both discriminative power and representativeness. Environments exhibiting both strong discrimination and representativeness were considered ideal for genotype evaluation. Notably, E5 and E2 were optimal test environments for DTF, PH, PBP, and 100-SW; E1, E2 and E3 for DTM; E3 and E1 for LPP and NSPP; E4 for SPP and PC; E4 and E3 for SYPP; E3 for Ca; E4 for P; E5 for Mg; E4 for Fe; and E2 and E4 for Zn. These environments proved effective for selecting broadly adapted genotypes due to their combined discriminative and representative qualities. In contrast, some environments displayed strong discriminative ability but poor representativeness. These included E3, E4 and E1 for DTF; E4 and E5 for DTM and LPP; E3 and E4 for PH and PBP; E1 and E4 for 100-SW; E4 and E5 for NSPP; E1 and E2 for SYPP; E1 and E5 for SPP; E4 and E2 for Ca; E5 for P, Fe and Zn; E1 for Mg; and E5 and E2 for PC.

#### Which-won-where pattern

**Fig. 7:**
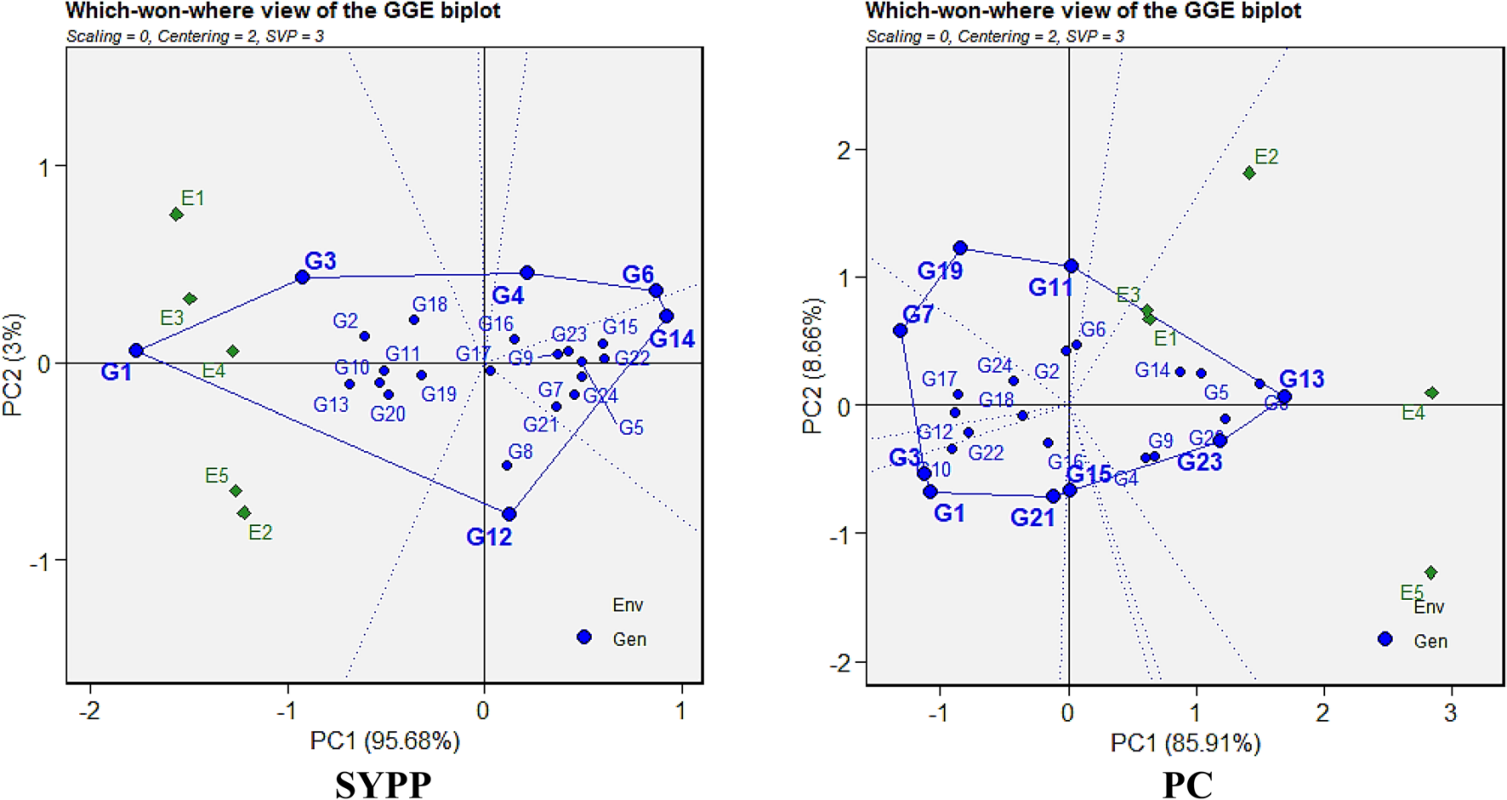
GGE (Genotype and Genotype × Environment Interaction) biplot of which-won-where pattern for SYPP and PC among 24 Tartary buckwheat genotypes under five test environments.

The which-won-where GGE biplot pattern for 24 Tartary buckwheat genotypes across five test environments is illustrated in Fig. 7 and Supplementary Fig. 5. The dotted lines from the centre of origin divided the polygon into 10 (P and Zn), 9 (PC and Ca), 8 (PBP and NSPP), 7 (PH and Fe), 6 (DTF, DTM, LPP, SPP and SYPP) and 5 (100-SW and Mg) sections. One mega-environment was observed for traits like DTF, DTM, LPP, PH, NSPP, SPP, SYPP, and PC; two for PBP, Mg, and Fe; three for 100-SW, Ca, and Zn; and four for P. Genotypes located at the polygon vertices were considered best performers in their respective sectors. Check G2, followed by G15, was found to be highly stable and early flowering across all five environments, making them consistent performers. For DTM, G24 and check G2 were the best performers in all five environments. Check G1 emerged as a stable and top-performing genotype under both systems for LPP, PH, SPP, and SYPP, along with G23 and G11 for LPP, and G12 for PH. In PBP, G23 was superior under E1, E2, E3 and E5, while G19 and G7 showed stability under E4. In 100-SW, G23 was best under all test environments except E4, while G24 and G12 performed better under E4. Genotypes such as G3 and G1 for SYPP and G13 and G23 for PC performed well across all test environments. For Ca, G19 (E1, E3) and G14 (E5), performed well under specific environments, while G6 and G13 were superior in both E4 and E2. In the case of P, G8 and G23 (E2), G6 (E3, E4), G19 and G18 (E5), and G5 (E1) performed well under all the test environments. For Mg and Fe, genotypes such as G22, G2, G19, and G8 (Mg), and G2, G19, and G18 (Fe) were stable and best performing under E2 to E5, but none showed consistent stability in E1. For Zn, G13 and G2 (E2, E4, E5) represented top performers, while G21 (E3) and G23, G9, and G10 (E1) also exhibited notable performance.

### Best linear unbiased predictor (BLUP)

**Fig. 8:**
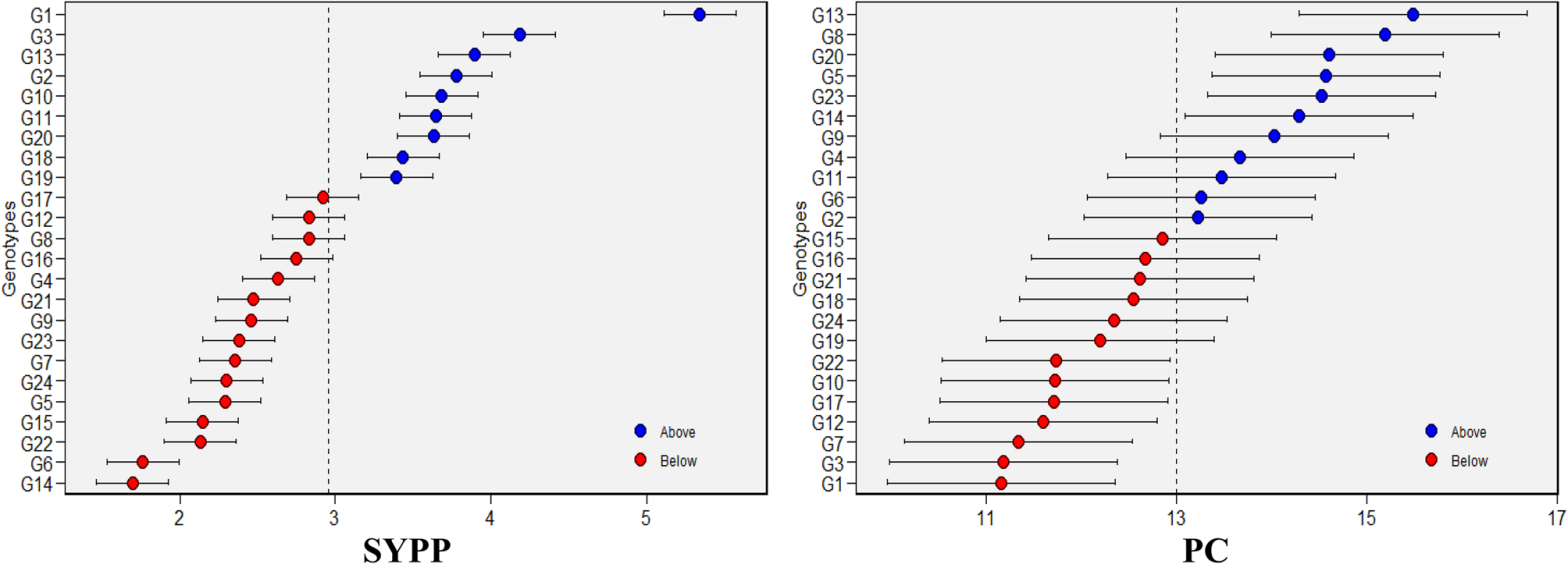
BLUP (Best Linear Unbiased Predictor) based predicted mean values for SYPP and PC in 24 Tartary buckwheat genotypes under five test environments.

Based on the likelihood ratio test for respective traits evaluated, eleven genotypes for DTF, 10 (DTM), 12 (LPP), 12 (PBP), 12 (PH), 14 (100-SW), 11 (NSPP), 13 (SPP), 9 (SYPP), 13 (Ca), 12 (P), 10 (Mg), 8 (Fe), 12 (Zn) and 11 (PC), had above-average predicted mean values (Fig. 8 and Supplementary Fig. 6). Among genotypes, check G1 had the highest above-average predicted value for trait LPP, PH, NSPP, SPP and SYPP; G12 for 100-SW; check G2 for DTF, DTM and Fe; G23 for PBP; G14 for Ca; G6 for P; G22 for Mg; and G13 for Zn and PC, respectively.

### MTSI

In our study, MTSI was computed based on both agronomic and nutritional parameters evaluated in 24 Tartary buckwheat genotypes. Out of fifteen, a total of six factors with an eigenvalue of more than 1.00, explaining 75.7% variation, were selected (Table 6). Factor 1 (FA1) accounted for 21.4% of the total variance and was primarily associated with negative loadings for Mg (− 0.887), Fe (− 0.828), and Zn (− 0.706), indicating that this factor represents the genotypes’ capacity for micronutrient accumulation. Factor 2 (FA2) explained 15.6% of the variance and was dominated by negative loadings for Ca (− 0.794), P (− 0.728), and PC (− 0.652), suggesting it captures variation in macronutrient partitioning and protein synthesis efficiency. Factor 3 (FA3) contributed 12.8% to the variance and was defined by strong negative associations with DTF (− 0.819) and DTM (− 0.792), as well as 100-SW (− 0.605), reflecting the influence of this factor on developmental timing and seed development. Since early flowering and maturity are often desirable, genotypes with lower FA3 scores were favoured in the ideotype construction. Factor 4 (FA4), responsible for 10.5% of the variance, was characterized by high positive loadings for NSPP (0.808) and SYPP (0.855), representing yield component traits and overall reproductive productivity. Factor 5 (FA5), explained 8.1% of the variance and was most strongly associated with PBP (− 0.907) and LPP (− 0.499), indicating its role in capturing variation related to plant branching architecture. Lastly, Factor 6 (FA6), which explained 7.3% of the variance, showed high positive loadings for LPP (0.805), PH (0.770), and SPP (0.751), suggesting this factor is related to plant stature development.

**Table 6:** Communality, factors, selection differential for 15 agronomic and nutritional parameters estimated in 24 Tartary buckwheat genotypes under five test environments during 2021–23 (E1-E5).

Trait	FA1	FA2	FA3	FA4	FA5	FA6	Comm	Uniq	Factor	Selection differential for the mean of the variables							Selection differential for WASSBY index			
										X_o_	X_s_	SD	SD%	SG	SG%	h^2^	X_o_	X_s_	SD	SD%
DTF	− 0.22	− 0.22	− 0.82	− 0.13	0.09	0.02	0.79	0.21	3	50.00	53.10	3.07	6.14	2.98	5.95	0.97	50.8	62.3	11.5	22.7
DTM	0.02	− 0.06	− 0.79	− 0.03	− 0.11	− 0.03	0.65	0.35	3	88.50	93.60	5.14	5.80	4.95	5.59	0.96	45	58.1	13.1	29
LPP	− 0.01	− 0.07	0.03	− 0.06	− 0.50	0.81	0.91	0.09	6	28.20	32.40	4.16	14.70	3.95	14.00	0.95	45.9	59.3	13.4	29.1
PBP	0.03	− 0.18	− 0.08	0.01	− 0.91	− 0.03	0.86	0.14	5	2.90	2.90	0.00	0.08	0.00	0.08	0.91	50.8	57	6.19	12.2
PH	− 0.22	0.03	− 0.01	0.19	0.04	0.77	0.68	0.32	6	86.80	96.20	9.45	10.90	9.28	10.70	0.98	42.5	57.2	14.6	34.4
100-SW	0.43	0.09	− 0.61	0.33	− 0.10	− 0.13	0.69	0.31	3	2.21	2.27	0.06	2.89	0.06	2.78	0.96	66.9	70.9	4.05	6.05
NSPP	− 0.12	0.04	0.13	0.81	0.37	0.21	0.86	0.14	4	123.00	137.00	14.40	11.70	14.00	11.40	0.98	45.6	58.2	12.7	27.8
SPP	0.01	− 0.12	0.05	0.06	0.18	0.75	0.62	0.38	6	7.89	8.17	0.28	3.54	0.25	3.16	0.89	40	43.7	3.65	9.12
SYPP	− 0.22	0.14	− 0.05	0.86	− 0.27	0.07	0.88	0.12	4	2.96	3.84	0.89	30.00	0.88	29.60	0.99	47.8	65.4	17.5	36.7
Ca	− 0.30	− 0.79	− 0.14	− 0.10	− 0.09	− 0.02	0.76	0.24	2	54.60	56.60	2.02	3.71	1.67	3.06	0.83	60.7	72.4	11.7	19.3
P	0.33	− 0.73	0.11	0.12	− 0.05	0.14	0.69	0.31	2	361.00	364.00	2.85	0.79	2.37	0.66	0.84	53	50.6	− 2.44	− 4.61
Mg	− 0.89	− 0.01	0.07	− 0.01	0.02	0.26	0.86	0.14	1	207.00	218.00	11.60	5.60	9.94	4.81	0.86	61.8	79.5	17.7	28.7
Fe	− 0.83	− 0.01	− 0.07	0.27	0.01	− 0.09	0.77	0.23	1	3.95	4.32	0.38	9.56	0.34	8.54	0.89	49.9	68.7	18.8	37.7
Zn	− 0.71	0.51	− 0.07	0.24	− 0.01	0.13	0.83	0.17	1	2.94	3.23	0.30	10.10	0.28	9.56	0.95	56.5	78.3	21.8	38.5
PC	0.09	− 0.65	− 0.22	− 0.15	− 0.10	0.08	0.52	0.48	2	13.00	12.90	− 0.06	− 0.46	− 0.05	− 0.40	0.86	47.3	45.9	− 1.33	− 2.82
Mean							0.76	0.24												

Comm.: Communality; Uniq.: Uniqueness; X_o_: overall; X_s_: selected; SD: Selection differential; SG: Selection gain; DTF: Days to 50% flowering; DTM: Days to 80% maturity; LPP: Leaves per plant; PBP: Primary branches per plant; PH: Plant height; 100-SW: 100 seed weight; NSPP: Number of seed per plant; SPP: Straw yield per plant; SYPP: Seed yield per plant; Ca: Calcium; P: Phosphorus; Mg: Magnesium; Fe: Iron; Zn: Zinc; PC: Protein content.

**Fig. 9:**
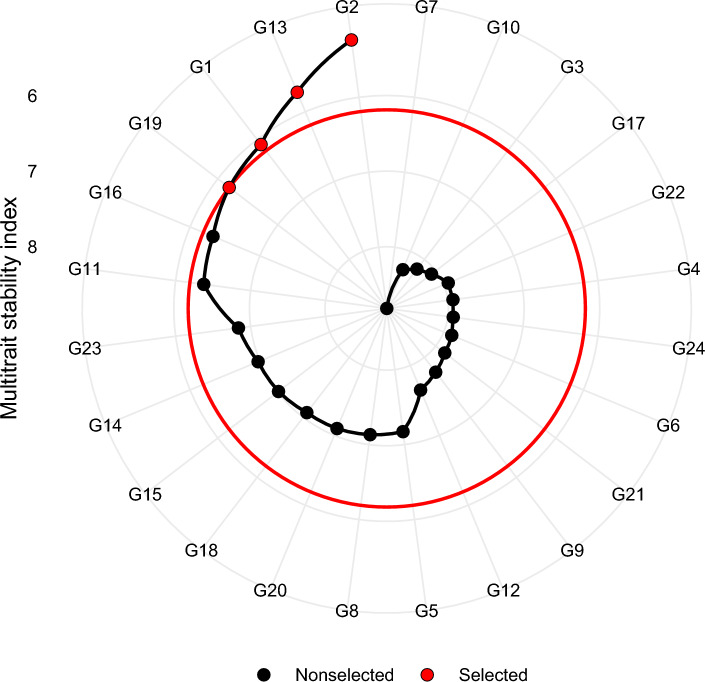
Multi-trait stability index (MTSI) of 24 Tartary buckwheat genotypes under five test environments.

The communality following varimax rotation ranged from 0.52 (PC) to 0.91 (LPP) with an average value of 0.76. The communalities for most traits were high, confirming that the six-factor model captured the majority of trait variance. These factor loadings were used to define the ideotype and calculate genotype-ideotype distances in the MTSI framework, accounting for the direction of trait desirability. Selection differentials based on these factors revealed that genotypes with high FA1 scores had substantial increase in micronutrient content, with selection gains of up to 37.7% for Fe and 38.5% for Zn. Similarly, selection for FA4 and FA6 led to notable improvements in yield components and plant stature, respectively. The MTSI values of the Tartary buckwheat genotypes are presented in Fig. 9. The genotypes determined in red colour dots were selected based on their MTSI values at 20% selection intensity. Hence, based on mean performance and stability, and following standard rounding practice (i.e., 4.8 ≈ 5) in breeding studies, the five most ideal Tartary buckwheat genotypes identified were check G2 (ranked first), followed by G13, check G1, G19, and G16.

## Discussion

Tartary buckwheat, an underutilized and largely neglected crop, is now gaining global attention for its high nutritional value, resilience, and potential to enhance food security and promote human health^24,25^. Hence, the identification of stable and high-yielding nutrient-rich Tartary buckwheat varieties that can thrive in diverse environments holds great promise for sustainable agriculture and global nutrition. Therefore, in our study, a total of 24 Tartary buckwheat genotypes, including two checks, were evaluated for nine agronomic and six nutritional parameters across five specific test environments (E1–E5), representing different management practices (natural and inorganic), locations, and seasons over a two-year period (2021–23). The trait evaluation across different environments revealed significant differences for all the evaluated agronomic and nutritional traits. Following our results, similar significant differences have been reported in *F. esculentum* and *F. tataricum* among 97 buckwheat genotypes for 16 traits^26^, and among 61 buckwheat genotypes for 17 traits using one-way analysis^27^. Also, to estimate the variation, pooled ANOVA was performed, which revealed significant variation among evaluated genotypes (G), environment (E) and their interaction (GEI) for almost all the characters, except for environmental variation estimated for P and Fe content. Similar significant findings were also reported for three buckwheat (*F. esculentum* Moench) cultivars in three environments for different agronomic and nutritional characteristics^28^. Likewise, significant GEI were also reported among buckwheat accessions (*F. Tataricum* Geartn)^29^. In a two-year study over two locations, a similar significant GEI was observed for six agronomic traits among 20 buckwheat genotypes (*F. Tataricum* Geartn and *F. esculentum* Moench), except for grains per plant and grain yield^30^. Further, Bartlett’s test showed non-significant K-squared values for all traits across five environments, indicating no major variance heterogeneity among environment means^23,31,32^.

Pooled mean performance and estimation of genetic parameters provided further insights into the potential and magnitude of variability in the evaluated genotypes^33^. Genotypes G13 and G8, along with check G1 (Shimla B1), were found superior for SYPP, LPP, PBP, PH, and PC, whereas G23 was found to be superior for LPP, PBP, 100-SW and SPP. Besides, genotypes G7, G13, and G19, along with both checks G1 and G2, were found promising for higher micronutrient concentration (Ca, P, Fe and Zn). Similar variations in micronutrients concentration (Ca: 49.2–57.6 mg/100 g, Mg: 194.5–216.5 mg/100 g; P: 346.6 to 375.2 mg/100 g; Fe: 3.5 to 4.2 mg/100 g; Zn: 2.6–3.2 mg/100 g) and protein content (9.8–11.3%) has been reported in Tartary buckwheat genotypes in this region^34,35^. Hence, the genotypes identified with superior nutrient profiles could thus contribute to biofortification strategies and help alleviate micronutrient deficiencies when incorporated into local diets. Additionally, high GCV and PCV, with high broad-sense heritability (h^2^_bs_) and high genetic advance as a percentage of the mean (GAM) were observed for NSPP and SYPP. Whereas high h^2^_bs_ and moderate GAM were observed for PH, moderate h^2^_bs_ and high GAM for LPP, and moderate h^2^_bs_ and GAM for DTF, 100-SW and Zn. Hence, these traits indicated their potential as selection criteria for screening high-yielding genotypes in future buckwheat breeding programs. These results align with similar findings in buckwheat^25,36–38^, pearl millet hybrids^39^, sesame^40^, and soybean^41^, where genetic parameters were crucial in identifying traits with high selection potential. Association analysis using Pearson’s correlation coefficients revealed significant positive correlation among agronomical traits, such as SYPP with NSPP and PH, SPP with LPP and PH. Whereas biochemical traits Fe and Zn showed a positive correlation with SYPP. Thus, selection of these characters may result in correlated responses in other characters, including seed yield. A similar relationship for agronomical traits in buckwheat genotypes has been reported in several studies^42–44^.

Multi-environment trials are instrumental in identifying stable and high-trait-performance genotypes in breeding programmes. In our study, the AMMI model revealed significant differences for genotypes, environment and GEI for almost all the traits, which demonstrated the substantial role of environmental variability in influencing genotype performance, supporting previous studies on crops sensitive to environmental fluctuations^45–47^. Partitioning the GEI through PCA highlighted the dominance of the first two IPCAs, which captured the majority of the interaction variability in common bean^48^, chickpea^49^ and barley^50^. The WAAS further enhanced the stability assessment by integrating all IPCAs, providing a comprehensive measure of genotype stability. It allowed simultaneous selection of genotypes based on high trait performance and stability. In the WAAS biplot, genotypes in quadrant IV, with high trait means and low WAAS scores, were stable and high-performing, making them ideal for general cultivation. In our study, check G1 (Shimla B1) showed higher stability and high mean performance for traits such as SYPP, PH, Zn, and PC, while check G2 (Himpriya) was found consistently good for SYPP, Mg, Ca, Fe, and NSPP. Besides, both genotypes G4 and G5 often appeared for traits LPP, PBP, PC, and Ca. Other genotypes viz*.,* G8, G13, G14, G18 and G19 showed repeated presence in nutritional traits (Zn, Fe, Mg, PC) and yield components (SPP, SYPP, 100-SW), whereas G11 and G12 performed well for 100-SW, SPP, and PC, while maintaining good stability. In particular, G13 and G19 along with both checks G2 and G1 stood out for nutritional quality with agronomic stability, indicating their utility in multi-trait selection programs. Similar findings have been reported in wheat^51^ and cotton^52^, where WAAS effectively identified stable, high-performing genotypes. Additionally, WAASBY index (score of 100) identified superior genotypes such as G15, G19, and G23 along with both checks G2 and G1 for various agro-morphological (DTF, DTM, PH, LPP, PBP, NSPP and SYPP and nutritional traits (Ca, Zn, P and PC). Similar approach for ranking of genotypes based on WAASBY index has been used in recently reported studies^23,53^.

GGE biplot are scatter plots based on environment-centered data and offers visual insights into genotype performance and stability by effectively separating the main genotype effects and GEI^17,54^. In mean vs stability biplot, the genotypes positioned closer to the average environment coordinate (AEC) were identified as stable and high performing^55,56^. The biplot revealed that genotypes G23 and both checks (G1 and G2) consistently excelled in multiple traits, with G1 showing high stability across eight traits (LPP, PH, NSPP, SPP, SYPP, Mg, Fe, and Zn) in both systems, and G2 and G23 performing well across several environments. G13 and G19 also showed strong, stable performance across traits like PC, Mg, Fe, Zn, and Ca, with G19 performing particularly well in all test environments except E4 environments. The distinguishing vs representativeness biplot illustrates an environment’s capacity to effectively differentiate genotypes based on their performance while also indicating how well it represents the overall mean performance across all tested environments^57^. The plot indicated that environments E2 and E5 were ideal for broad genotype evaluation, while the environment E4 was crucial for selecting genotypes suited to sustainable cultivation. Genotypes such as G13, G23, and G19 along with both checks G2 and G1 performed consistently across both systems. The which-won-where biplot in GGE analysis identifies the best-performing genotypes in specific environments by forming a polygon with vertex genotypes, which are superior in their respective sectors. The biplot identified G2 and G15 as stable and early-flowering across all five test environments (E1-E5). Check G1 excelled in LPP, PH, SPP, and SYPP, while G23 and G12 stood out in specific traits. G23 performed well for PBP and 100-SW under E1, E2, E3 and E5 while G19 excelled in P, Fe, and Zn under E4. Genotypes G3, G13, and G6 showed consistent performance highlighting their suitability for multi-environment selection. Similar applications of GGE biplots have been reported in buckwheat^29^, rice^58^ and barley^50^.

While the GGE biplot offers insights into genotype stability and performance, environmental effects can often obscure genotypic effects, complicating selection. The BLUP model, by accounting for such variability, proved effective in identifying stable and high-performing genotypes. By treating genotypic effects as random, BLUP accounted for environmental heterogeneity, enhancing prediction accuracy^59^. Genotypes with high BLUP values, such as G1, demonstrated consistent stability and performance for traits SYPP, SPP and quality attributes like PC and Zn, reinforcing BLUP’s effectiveness in predicting stable genotypes, as observed in other crops like rice^60^, wheat^61^ and pea^62^. Moreover, several tools facilitate the simultaneous selection of genotypes based on multiple traits. One such tool, the Multi-Trait Stability Index (MTSI), was utilized in our study to identify and rank genotypes that demonstrate both stability and desirable trait performance^21,22^. The MTSI approach proved effective in integrating multiple traits and identifying genotypes aligned with the desired ideotype. The factor structure revealed biologically meaningful groupings, allowing selection based on trait desirability rather than isolated performance. This multivariate selection strategy strengthens breeding decisions by prioritizing genotypes that combine nutritional quality with agronomic adaptability, supporting their advancement in varietal development. A similar approach for ranking genotypes based on multiple traits has been used in soybean^63^, wheat^64^ and barnyard millet^65^.

## Conclusion

The present study provides a detailed analysis of GEI in 24 Tartary buckwheat genotypes tested across five test environments using different advanced statistical models. The findings highlight the significant variability for all the traits in five test environments. Variability estimates showed that traits like SYPP, NSPP, PH, LPP, DTF, 100-SW and Zn can be utilized for yield improvement and screening high-yielding buckwheat genotypes. Based on mean performance, WAAS, WAASBY index, GGE biplots and BLUP, genotypes G13, G19, G15 and G23, along with both checks (G1 and G2) were identified as the most stable and high-performing selections across multiple traits. Additionally, G1 was identified as the top performer for traits like NSPP and SYPP, while G13 consistently excelled in PC. Also, E2, E5 and E4 were the most discriminative and representative environments for overall stability assessments. Moreover, selection index, i.e. MTSI, also identified G13 and G19 along with both checks, G2 and G1, as the most ideal genotypes, showcasing superior stability and performance across various traits. These genotypes show strong potential for cultivation across diverse environments and serve as promising parental lines in breeding programs targeting nutritionally rich and agro-adapted Tartary buckwheat cultivars. Overall, these findings support the development of resilient, nutrient-dense buckwheat varieties to improve both crop performance and food quality.

## Materials and methods

### Experimental material

Healthy seeds with uniform size, shape and moisture content (~ 12%) of 24 Tartary buckwheat genotypes, including two checks, viz*.,* Shimla B1 and Himpriya, were selected for the study. The details of the selected genotypes used, along with their source, are mentioned in Table 7.

**Table 7:** Details of the 24 Tartary buckwheat genotypes used in the study.

Code	Genotypes	Source	Code	Genotypes	Source
G1	Shimla B1 (C)	NBPGR, Shimla	G13	IC 341,589	NBPGR, Shimla
G2	Himpriya (C)	CSKHPKV, Palampur	G14	IC 356,112	NBPGR, Shimla
G3	IC 26,755	NBPGR, Shimla	G15	IC 37,288	NBPGR, Shimla
G4	Sangla B 444	CSKHPKV, Palampur	G16	IC 341,667	NBPGR, Shimla
G5	Sangla B 214	CSKHPKV, Palampur	G17	IC 345,059	NBPGR, Shimla
G6	Sangla B 129	CSKHPKV, Palampur	G18	IC 323,723	NBPGR, Shimla
G7	Sangla B 5	CSKHPKV, Palampur	G19	IC 341,674	NBPGR, Shimla
G8	IC 46,160	NBPGR, Shimla	G20	IC 341,683	NBPGR, Shimla
G9	Himgiri 109,728	NBPGR, Shimla	G21	IC 371,665	NBPGR, Shimla
G10	IC 318,859	NBPGR, Shimla	G22	IC 42,430	NBPGR, Shimla
G11	IC 109,729	NBPGR, Shimla	G23	EC 286,377	NBPGR, Shimla
G12	IC 47,929	NBPGR, Shimla	G24	Chitkul	Local

C: Check; NBPGR: National Bureau of Plant Genetic Resources; CSKHPKV: Chaudhary Sarwan Kumar Krishi Vishvavidyalaya; Local: Genotypes from local sources.

### Experimental site, layout and methodology

The experiment was carried out by generating five environments (E1-E5) *i.e. kharif* (May–August) 2021 under inorganic production system at Mountain Agricultural Research and Extension Centre (MAREC), Sangla, Himachal Pradesh (H.P.), India (E1), *rabi* 2021–22 (February-April) at Palampur, H.P. under inorganic production system (E2), *kharif* 2022 under inorganic production system at MAREC, Sangla, H.P. (E3), *rabi* 2022–23 at Palampur, H.P. under natural farming production system (E4) and during *rabi* 2022–23 at Palampur under inorganic production system (E5). The details of the different test locations over the two locations are provided in Table 8 and Supplementary Fig. 7. A total of 24 genotypes, including two checks, were evaluated in Randomized Block Design (RBD) with 3 replications. Each genotype was planted in two rows of 3 m length, having 30 cm row to row and 10 cm plant to plant distance. Standard crop management practices were followed except in E4, where the typical fertilizer dosage for buckwheat includes 40 kg/ha of nitrogen (N), 20 kg/ha of phosphorus (P), and 20 kg/ha of potassium (K), with half the total dosage applied at sowing and the remainder during the clipping/flowering of the crop. In E4, the soil was first treated with *jeevamrit* (10%) and seeds with *beejamrit* before sowing. Vermicompost was applied at 5 t/ha at the time of sowing, and organic liquid manure (vermiwash at 10%) was sprayed every 15 days. For all environments, irrigation was scheduled once a week or every 10 days, depending on weather conditions, with reliance on natural rainfall and supplemental irrigation when necessary. All recommended agronomic management and package of practices were followed to ensure healthy crop growth.

**Table 8:** Details of different locations, its average annual weather parameters and soil type.

Sr. no	Parameters	Location code				
		E1	E2	E3	E4	E5
1	Cropping period	May-Aug, 2021	Feb-April, 2022	May-Aug, 2022	Feb-April, 2023	Feb-April, 2023
2	Farming System	Inorganic	Inorganic	Inorganic	Natural faming (ZBNF)	Inorganic
3	Location (H.P.)	Sangla	Palampur	Sangla	Palampur	Palampur
4	Latitude	31° 29′ N	32° 6′ N	31° 29′ N	32° 6′ N	32° 6′ N
5	Longitude	78° 60′ E	76° 3′ E	78° 60′ E	76° 3′ E	76° 3′ E
6	Altitude (a.m.s.l.)	2621	1219	2621	1219	1219
7	Agro-climatic zone	Mid-hill temperate	Mid-hill sub humid	Mid-hill temperate	Mid-hill sub humid	Mid-hill sub humid
8	Average minimum temperature (℃)	10.10	11.83	10.50	12.00	12.00
9	Average maximum temperature (℃)	22.50	21.70	23.80	23.00	23.00
10	Total rainfall (mm)	625.70	1578	436.4	1578	1578
11	Relative humidity (%)	73.56	68.82	79.36	67.51	67.51
12	Soil texture	Sandy loam	Silty clay loam	Sandy loam	Silty clay loam	Silty clay loam
13	Soil pH	6.5–6.9	5.23–6.1	6.5–6.9	5.12–5.86	5.23–6.1

H.P.: Himachal Pradesh; ZBNF: Zero Budget Natural Farming.

Data was recorded on ten random plants per plot for nine quantitative agronomic traits namely, days to 50% flowering (DTF), days to 80% maturity (DTM), leaves per plant (LPP), plant height (PH), primary branches per plant (PBP), 100 seed weight (100-SW), number of seed per plant (NSPP), straw yield per plant (SPP) and seed yield per plant (SYPP). Simultaneously, various nutritional parameters such as calcium (Ca), phosphorus (P), iron (Fe), magnesium (Mg), zinc (Zn) and protein content (PC) were also measured. Mineral concentrations of Ca, Fe and Zn in all the genotypes were estimated using diacid method as suggested by Piper^66^. Mg content was estimated as per Sahrawat^67^ and P content was determined using vanado-molybdo-phosphoric acid yellow colour method^68^. Protein content was estimated using micro-kjeldahl method as described by Jackson^69^. The collection and evaluation of Tartary buckwheat germplasm were carried out in accordance with institutional guidelines, and all necessary permissions were obtained from the relevant authorities.

### Statistical analysis

Variation among genotypes for different traits were tested using analysis of variance (ANOVA) as per Panse and Sukhatme^70^ and the nature of differences among genotypes using post hoc test i.e. Duncan’s multiple range test (DMRT)^71^. Various variability parameters were also estimated such as genotypic coefficient of variation (GCV) and phenotypic coefficient of variation (PCV), heritability-broad sense (h^2^_bs_) and genetic advance as percent mean (GAM) as per Burton and De Vane^72^, Hanson et al.^72^ and Johnson et al.^73^, respectively. Bartlett’s test was further utilized to test the homogeneity of significant variation among genotypes for different traits. The effect of genotype, environment and genotype-by-environment interaction was then tested using mixed-effect model, considering genotypes as fixed factors and environments as random factors.

#### AMMI analysis

A variance analysis based on the AMMI model was performed for each trait to evaluate the stability of 24 Tartary buckwheat genotypes across five different environments. Equation (1) was used to perform stability analysis by the AMMI method^74^.

1 $$Y_{ge} = \mu + \alpha_{g} + \beta_{e} + \sum\limits_{n} {\lambda_{n} \gamma_{gn} \delta_{en} + \rho_{ge} }$$

where Y_ge_ is the yield of genotype g in environment e; µ is the grand mean; α_g_ is the genotype deviation from the grand mean; β_e_ is the environment deviation; ${\lambda }_{n}$ is the singular value for IPC_n_ and correspondingly ${\lambda }_{n}^{2}$ is its eigenvalue; γ_gn_ is the eigenvector value for genotype g and component n; δ_en_ is the eigenvector value for environment e and component n, with both eigenvectors scaled as unit vectors; and ρ_ge_ is the residual. The IPCs were obtained for each genotype and environment from AMMI analysis.

#### WAAS, BLUP and genotypic stability index estimation

WAAS were estimated from the IPCs of AMMI analysis of variance. The IPC1 in the traditional AMMI1 biplot was replaced by WAAS^75^.

2 $${WAAS}_{i}=\frac{{\sum }_{k=1}^{P}\left|{IPCA}_{ik}\times {EP}_{k}\right|}{{\sum }_{k=1}^{P}{EP}_{k}}$$

where WAAS_i_ is the weighted average of absolute scores of the *i*th genotype or environment; IPCA_ik_ is the absolute score of the *i*th genotype or environment in the *k*th IPC; and *EP*_*k*_ is the magnitude of the variance explained by the *k*th IPC.

A linear mixed model, i.e. BLUP (best linear unbiased predictor), was employed for evaluating the stability of genotypes across the environments. The variance components were estimated by restricted maximum likelihood. The estimations were performed assuming genotype and GEI as random effects. The significance of random effects was tested by the likelihood ratio test^75^. The BLUP of *i*th genotype was predicted as the sum of the general mean across overall environments and the genotypic effect. WAASB were estimated based on a single value decomposition of the GEI effects from the matrix of the BLUP. The equation of WAASB (3) is just like WAAS. The WAASBY was calculated, allowing weighting between its mean performance and stability based on Eq. (4):

3 $${WAASB}_{i}=\frac{{\sum }_{k=1}^{P}\left|{IPCA}_{ik}\times {EP}_{k}\right|}{{\sum }_{k=1}^{P}{EP}_{k}}$$

4 $${WAASBY}_{i}=\frac{\left({rG}_{g}\times {\theta }_{Y}\right)+\left({rW}_{g}\times {\theta }_{S}\right)}{{\theta }_{Y}+ {\theta }_{S}}$$

where (Eq. 3) *WAASB*_*i*_ is the weighted average of absolute scores of the *i*th genotype or environment; *IPCA*_*ik*_ is the absolute score of the *i*th genotype or environment in the *k*th IPC; and *EP*_*k*_ is the magnitude of the variance explained by the *k*th IPC. In Eq. (4), *WAASBY*_*i*_ is the superiority index with different weights between yield and stability for the *g*th genotype; *θ*_*Y*_ and *θ*_*S*_ are the weights for yield and stability, respectively; *rG*_*g*_ and rW_g_ are the rescaled values of the *g*th genotype for yield and WAASB, respectively.

#### GGE biplot analysis

GGE biplot analysis was done based on single value decomposition according to Eq. (5) ^16,76^.

5 $${Y}_{ij}-\upmu -{\upbeta }_{j}={\lambda }_{1}{\upxi }_{i1}{\upeta }_{j2}+{\upvarepsilon }_{ij}$$

where, *Y*_*ij*_ is the mean of *i*th genotype in *j*_*g*_ environment, *µ* is the mean of all genotypes, *β*_*j*_ is the main effect of *j*th environment, ${\varvec{\lambda}}$_1_ and ${\varvec{\lambda}}$_2_ are the special quantities for the first and second components, respectively, ξ_i1_ and ξ_i2_ are the special vectors of genotypes, and *η*_*j1*_ and *η*_*j2*_ are the environmental vectors of first and second components, respectively, and *ε*_*ij*_ is the remaining quantity for the *i*th genotype in *j*th environment.

#### MTSI

After rescaling the data, based on the genotype–ideotype distance, multi-trait stability index (MTSI) was utilized to select genotypes based on stability and mean performance simultaneously for multiple traits^21^. MTSI was computed as per Eq. (6).

6 $${MTSI}_{i}={\left[\sum_{j=1}^{f}{\left({\upgamma }_{ij}-{\upgamma }_{j}\right)}^{2}\right]}^{0.5}$$

where *MTSI*_*i*_ is the multi-trait stability index of the genotype *i*, *γ*_*ij*_ is the score of the genotype *i* in the factor *j*, and *γ*_*j*_ is the score of the ideal genotype in the factor *j*. The ideotype represents an ideal genotype that exhibits the most desirable values for all traits, serving as a benchmark for comparison.

The proportion of the MTSI of the *i*th genotype explained by the *j*th factor (ω_ij_) is used to show the strengths and weaknesses of genotypes/treatments and is computed using Eq. 7

7 $${\upomega }_{ij}= \frac{\sqrt{{D}_{ij}^{2}}}{{\sum }_{j=1}^{f}\sqrt{{D}_{ij}^{2}}}$$

where *D*_*ij*_ is the distance between the ith genotype and the ideotype for the jth factor. Low contributions of a factor indicate that the traits within such a factor are close to the ideotype. Factor grouping clusters correlated traits into latent components, simplifying data structure and enhancing the efficiency of selecting genotypes with superior multi-trait performance.

The statistical procedures viz*.,* AMMI, WAAS, WAASBY, BLUP and MTSI were analyzed using ‘*metan*’ package^77^ in R software ver. 4.3.2 (https://posit.co/).

## Supplementary Information

Supplementary Information 1.

Supplementary Information 2.

## Data Availability

All data generated or analysed during this study are included in this published article [and its supplementary information file].
