# Supplementary material for: Multi-environment evaluation and identification of Tartary buckwheat (Fagopyrum tataricum Gaertn.) genotypes for superior agronomic and nutritional potential in the North-Western Himalayas
Source: Sci Rep. 2025 Aug 22;15:30900. doi: 10.1038/s41598-025-15790-3 (PMC12373768; doi:10.1038/s41598-025-15790-3)
Supplement: Supplementary file 1 — Supplementary Information 1. [file 41598_2025_15790_MOESM1_ESM.pdf]

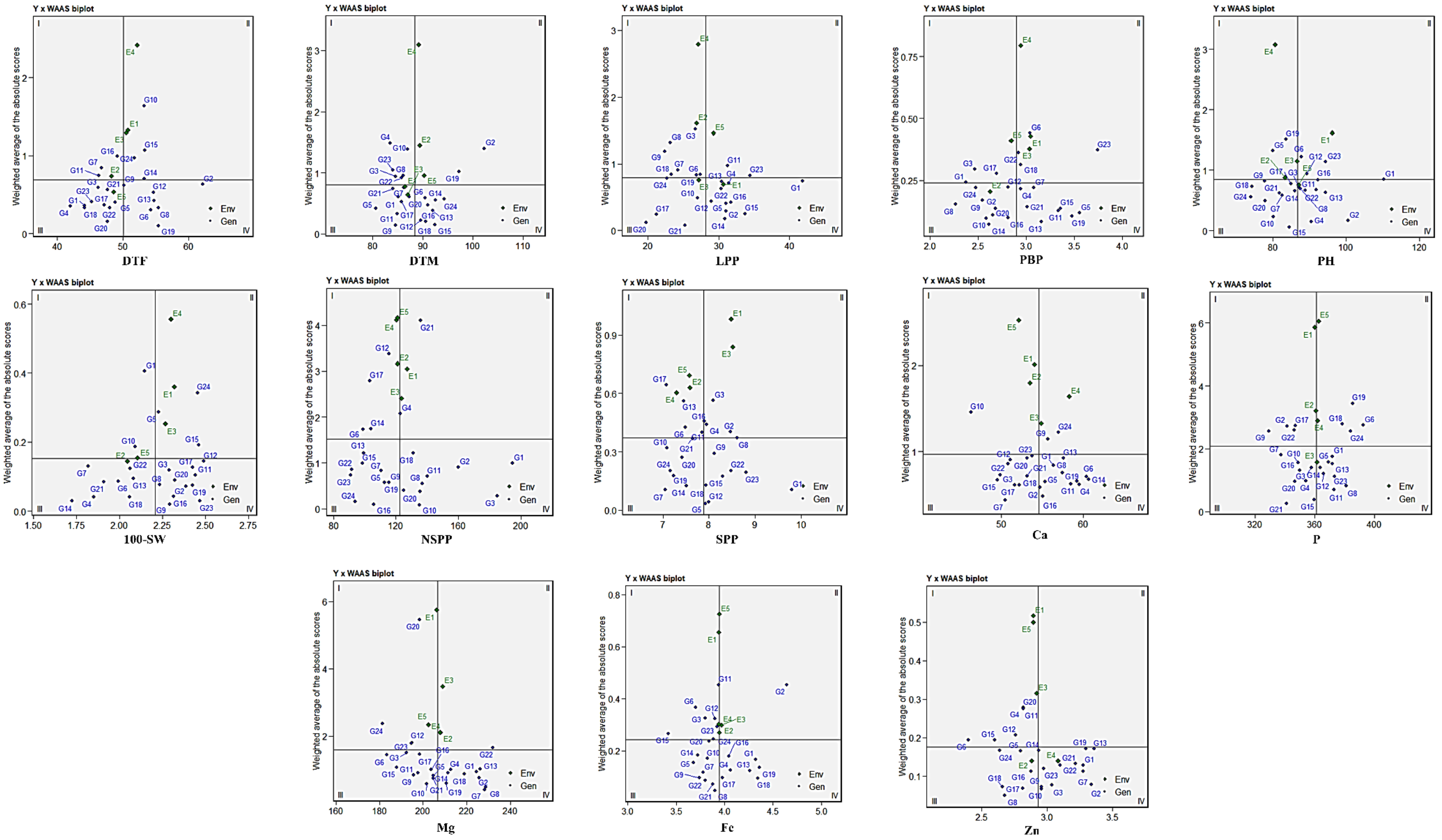

Supplementary figure 1: Mean × WAAS (Weighted Average of Absolute Scores) biplot of 24 Tartary buckwheat genotypes under five environments (DTF: Days to 50% flowering; DTM: Days to 80% maturity; LPP: Leaves per plant; PBP: Primary branches per plant; PH: Plant height; 100-SW: 100 seed weight; NSPP: Number of seed per plant; SPP: Straw yield per plant; Ca: Calcium; P: Phosphorus; Mg: Magnesium; Fe: Iron; Zn: Zinc).

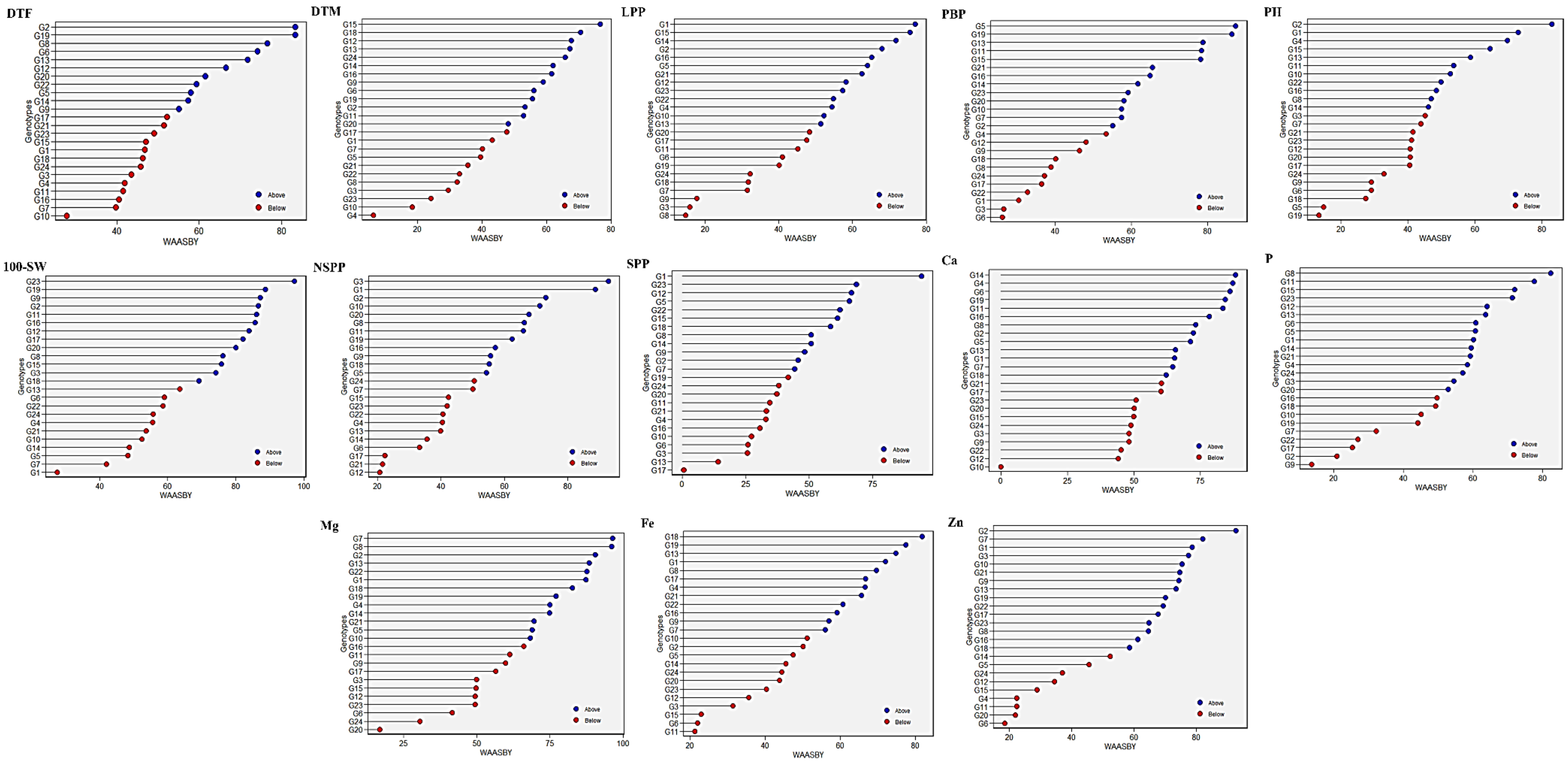

Supplementary figure 2: WAASBY (Weighted Average of Absolute Scores and Yield-Based Stability Index) based genotype ranking of 24 Tartary buckwheat genotypes under five environments (DTF: Days to 50% flowering; DTM: Days to 80% maturity; LPP: Leaves per plant; PBP: Primary branches per plant; PH: Plant height; 100-SW: 100 seed weight; NSPP: Number of seed per plant; SPP: Straw yield per plant; Ca: Calcium; P: Phosphorus; Mg: Magnesium; Fe: Iron; Zn: Zinc).

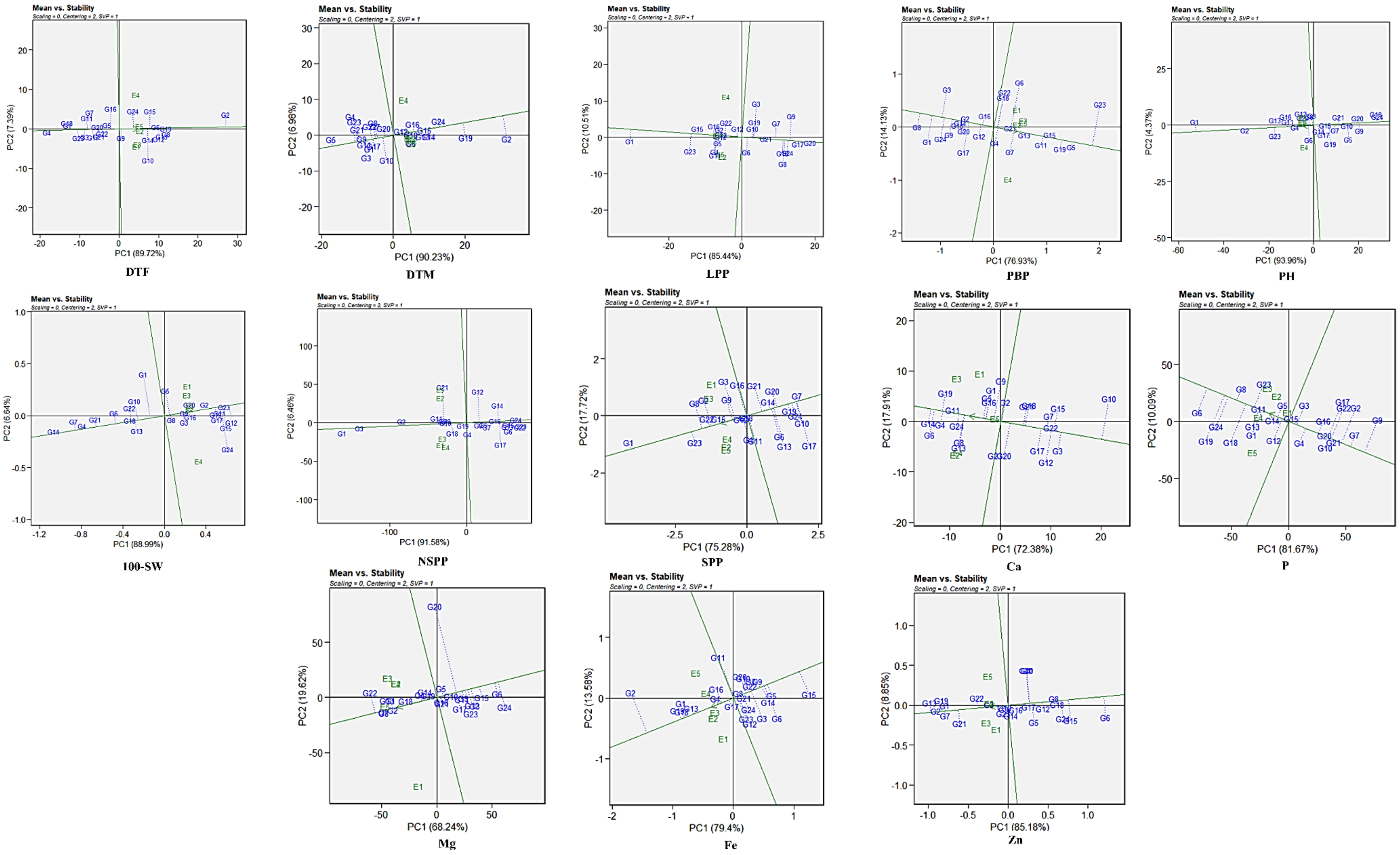

Supplementary figure 3: GGE (Genotype and Genotype × Environment Interaction) biplot of *mean vs stability* pattern of 24 Tartary buckwheat genotypes under five environments (DTF: Days to 50% flowering; DTM: Days to 80% maturity; LPP: Leaves per plant; PBP: Primary branches per plant; PH: Plant height; 100-SW: 100 seed weight; NSPP: Number of seed per plant; SPP: Straw yield per plant; Ca: Calcium; P: Phosphorus; Mg: Magnesium; Fe: Iron; Zn: Zinc).

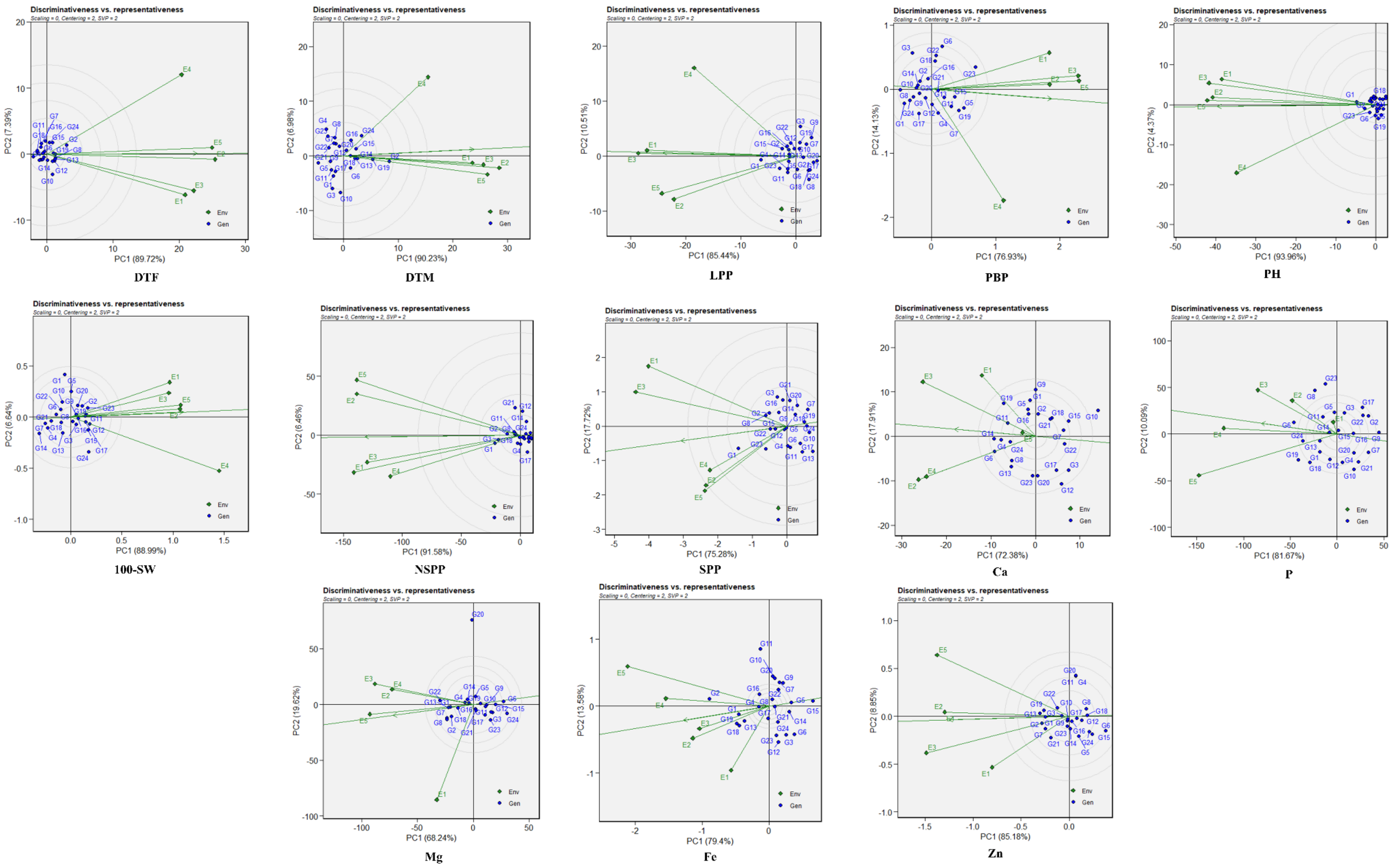

Supplementary figure 4: GGE (Genotype and Genotype × Environment Interaction) biplot of *discriminativeness vs representativeness* pattern of 24 Tartary buckwheat genotypes under five environments (DTF: Days to 50% flowering; DTM: Days to 80% maturity; LPP: Leaves per plant; PBP: Primary branches per plant; PH: Plant height; 100-SW: 100 seed weight; NSPP: Number of seed per plant; SPP: Straw yield per plant; Ca: Calcium; P: Phosphorus; Mg: Magnesium; Fe: Iron; Zn: Zinc).

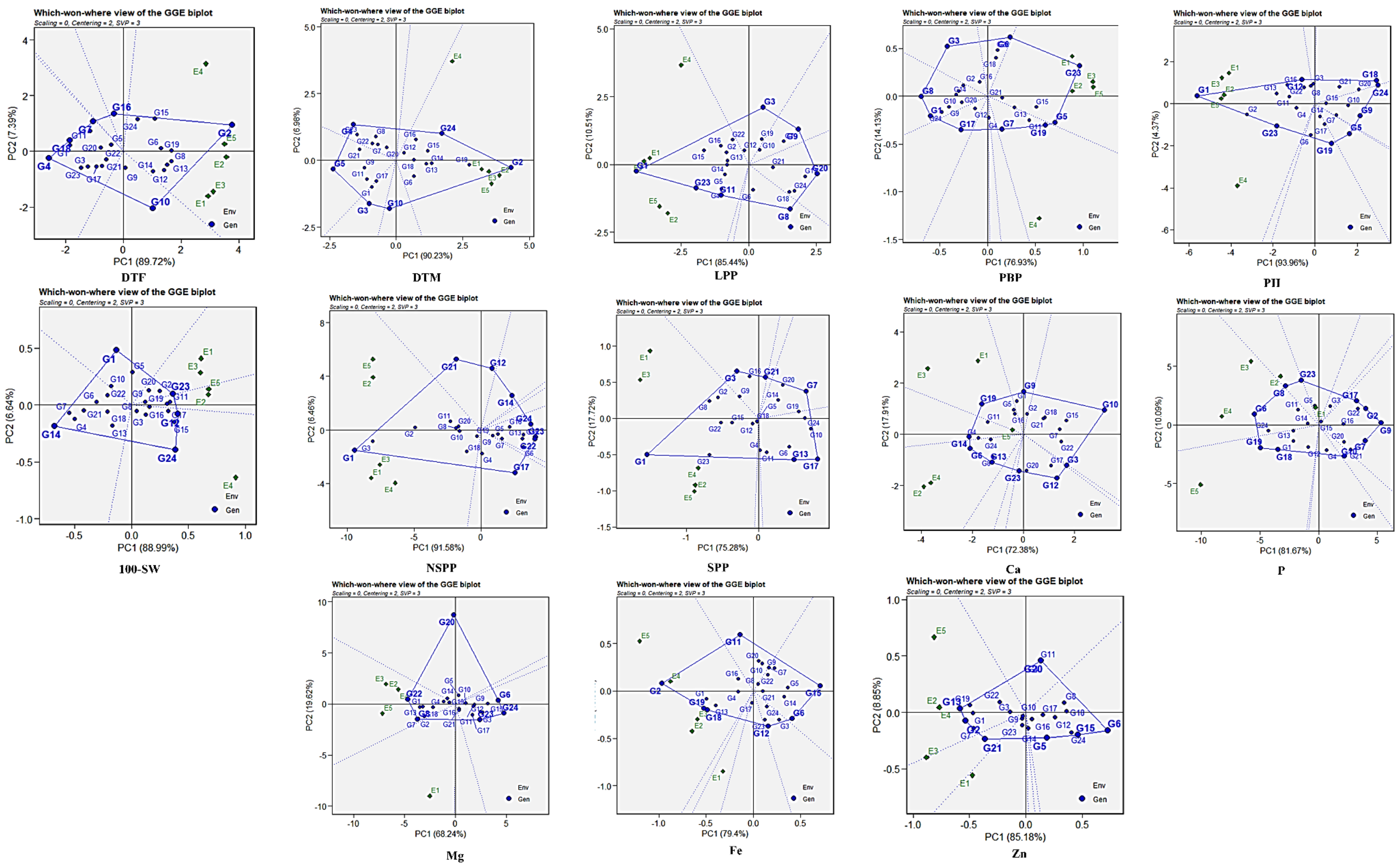

Supplementary figure 5: GGE (Genotype and Genotype × Environment Interaction) biplot of *which-won-where* pattern of 24 Tartary buckwheat genotypes under five environments (DTF: Days to 50% flowering; DTM: Days to 80% maturity; LPP: Leaves per plant; PBP: Primary branches per plant; PH: Plant height; 100-SW: 100 seed weight; NSPP: Number of seed per plant; SPP: Straw yield per plant; Ca: Calcium; P: Phosphorus; Mg: Magnesium; Fe: Iron; Zn: Zinc).

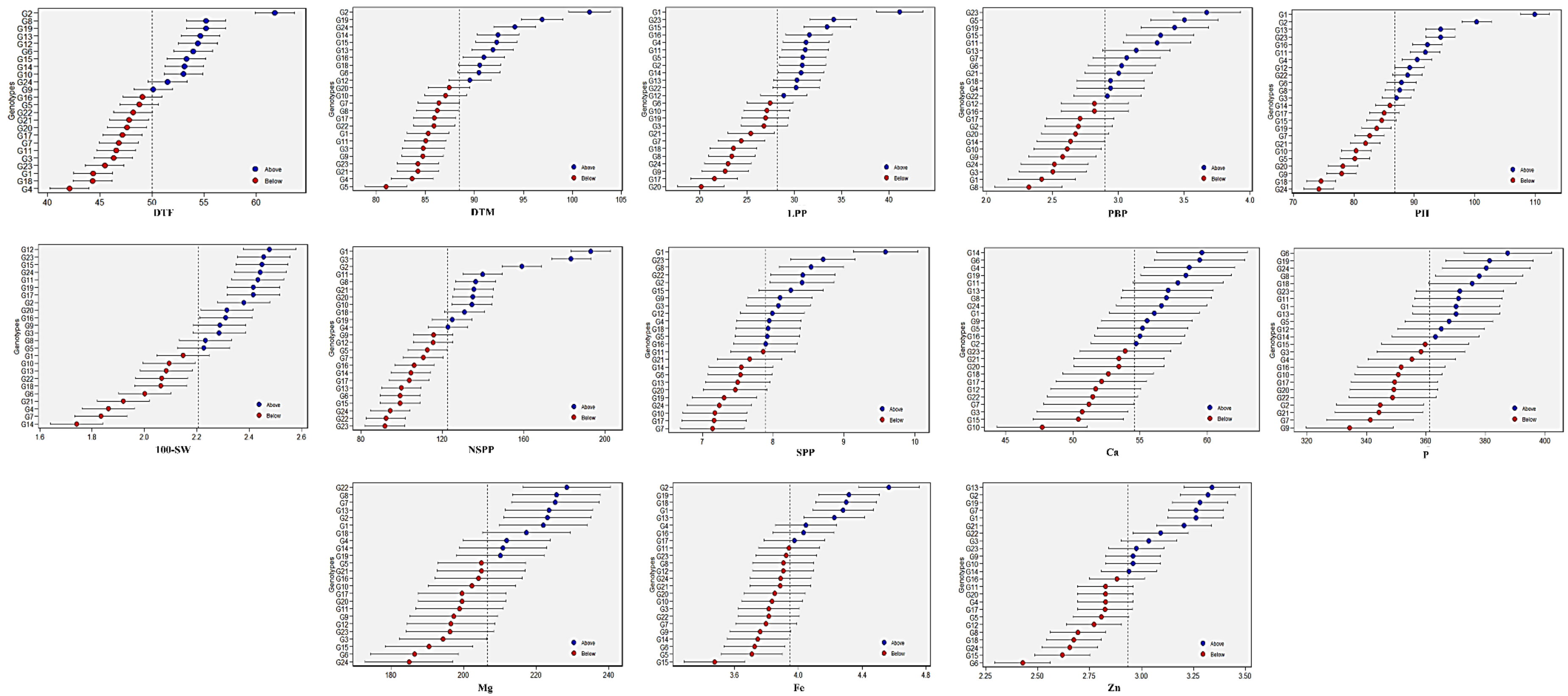

Supplementary figure 6: BLUP (Best Linear Unbiased Predictor) based predicted mean values of in 24 Tartary buckwheat genotypes under five environments (DTF: Days to 50% flowering; DTM: Days to 80% maturity; LPP: Leaves per plant; PBP: Primary branches per plant; PH: Plant height; 100-SW: 100 seed weight; NSPP: Number of seed per plant; SPP: Straw yield per plant; Ca: Calcium; P: Phosphorus; Mg: Magnesium; Fe: Iron; Zn: Zinc).

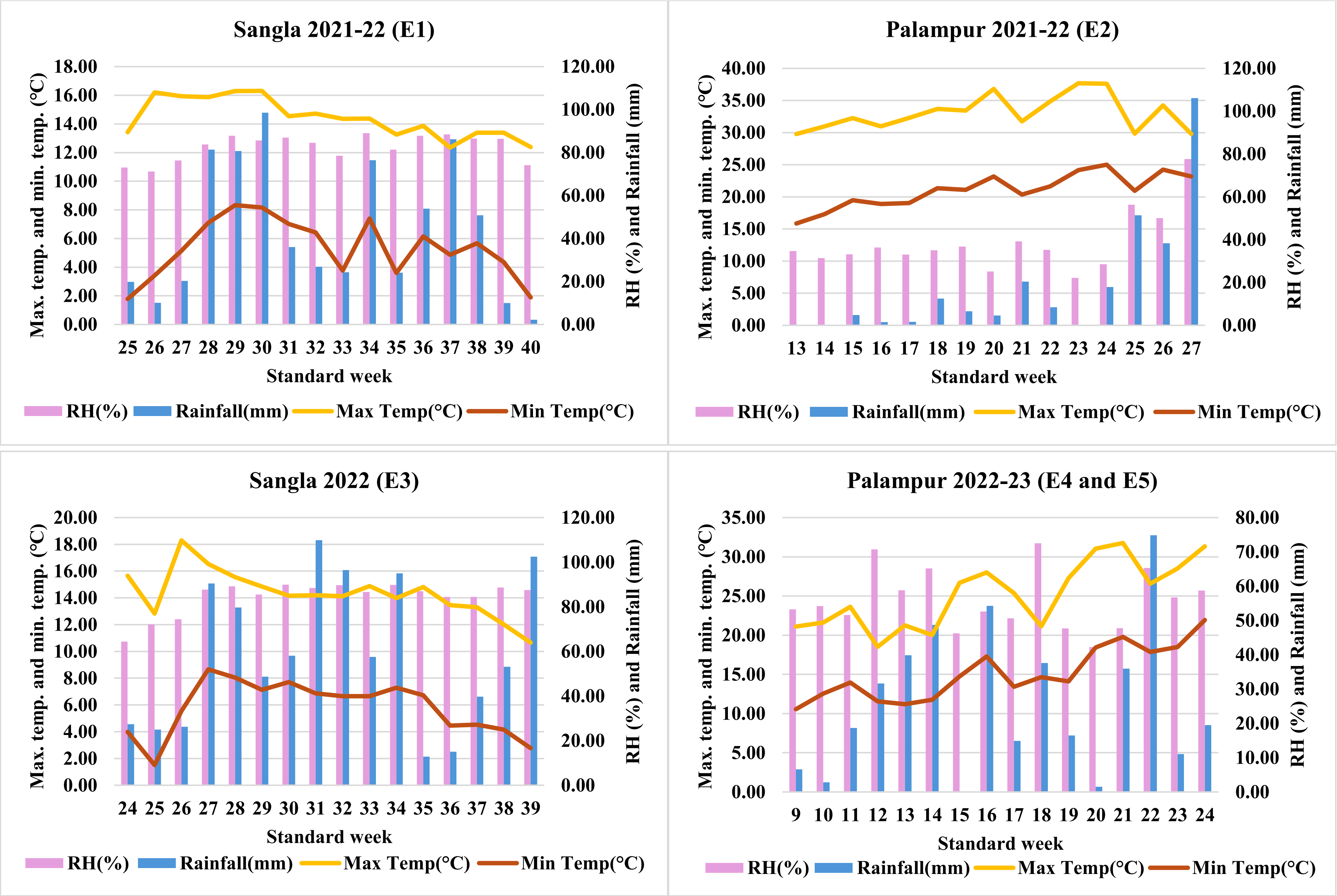

Supplementary figure 7: Seasonal meteorological profiles of the five test environments (E1 to E5) during the crop growth period. The graphs display maximum and minimum temperatures (°C), relative humidity (%), and total rainfall (mm) recorded weekly at each location.
