# Supplementary material for: Multi-environment evaluation and identification of Tartary buckwheat (Fagopyrum tataricum Gaertn.) genotypes for superior agronomic and nutritional potential in the North-Western Himalayas
Source: Sci Rep. 2025 Aug 22;15:30900. doi: 10.1038/s41598-025-15790-3 (PMC12373768; doi:10.1038/s41598-025-15790-3)
Supplement: Supplementary file 2 — Supplementary Information 2. [file 41598_2025_15790_MOESM2_ESM.pdf]

Supplementary table 1: Analysis of variance for all 15 agronomic and nutritional traits observed in 24 Tartary buckwheat genotypes

Source	d.f.	DTF	DTM	LPP	PBP	PH	100-SW	NSPP	SPP	SYPP	Ca	P	Mg	Fe	Zn	PC
**E1**																
**Rep**	2	2.625	2.042	0.676	0.027	6.86	0.004	0.69	0.044	0.02	0.19	13.291	47.762	0.005	0.002	0.276
**G**	23	62.73*	78.46*	101.42*	0.55*	206.20*	0.15*	2,771.69*	2.61*	3.17*	50.92*	211.90*	1,112.64*	0.17*	0.14*	1.84*
**Res**	46	1.509	5.288	0.751	0.013	5.957	0.003	10.133	0.033	0.01	2.235	95.945	30.213	0.008	0.006	0.114
**E2**																
**Rep**	2	1.764	11.375	0.126	0.007	1.993	0.003	3.719	0.016	0.007	2.324	62.277	33.351	0.007	0.006	0.035
**G**	23	87.91*	110.33*	75.17*	0.50*	221.35*	0.16*	2,676.32*	1.17*	1.98*	103.74*	666.77*	796.61*	0.23*	0.23*	8.01*
**Res**	46	1.416	5.621	0.452	0.014	3.763	0.003	11.106	0.038	0.008	1.695	126.624	21.439	0.008	0.008	0.085
**E3**																
**Rep**	2	0.792	5.056	0.01	0.047	7.049	0.001	10.518	0.019	0.005	0.258	12.399	11.225	0.004	0.004	0
**G**	23	70.28*	86.46*	110.55*	0.75*	232.76*	0.13*	2,330.50*	2.77*	2.83*	108.59*	1,343.57*	1,281.54*	0.21*	0.33*	1.86*
**Res**	46	2.299	4.766	0.497	0.012	5.532	0.003	9.097	0.06	0.008	2.279	88.234	22.204	0.011	0.008	0.119
**E4**																
**Rep**	2	0.264	3.292	0.13	0	9.592	0.001	3.311	0.003	0.021	4.807	76.93	14.565	0.001	0.04	0.009
**G**	23	73.93*	58.34	80.86*	0.56*	196.31*	0.31	1,867.04*	1.13	2.09*	90.10	2,004.77*	796.60*	0.33*	0.23*	20.66*
**Res**	46	2.032	5.422	0.361	0.014	4.418	0.003	8.374	0.043	0.009	2.384	85.109	29.794	0.012	0.005	0.121
**E5**																
**Rep**	2	2.625	3.014	1.869	0.021	3.609	0.003	16.709	0.007	0	7.863	1.676	76.016	0.011	0.005	0.034
**G**	23	84.44*	93.18*	87.08*	0.77*	234.48*	0.16*	2,805.43*	1.27*	2.09*	22.14*	3,129.17*	1,381.70*	0.63*	0.31*	21.44*
**Res**	46	2.147	4.854	0.748	0.013	5.155	0.004	12.506	0.041	0.009	1.648	97.673	26.22	0.01	0.004	0.109

All numeric columns are Mean sum of squares.

Rep: Replication; G: Genotypes; Res: Residual; E1-E5: Environments; DTF: Days to 50% flowering; DTM: Days to 80% maturity; LPP: Leaves per plant; PBP: Primary branches per plant; PH: Plant height; 100-SW: 100 seed weight; NSPP: Number of seed per plant; SPP: Straw yield per plant; SYPP: Seed yield per plant; Ca: Calcium; P: Phosphorus; Mg: Magnesium; Fe: Iron; Zn: Zinc; PC: Protein content

Supplementary table 2.1: Comparative pooled mean performance observed in 24 Tartary buckwheat genotypes for DTF, DTM and LPP during 2021-23.

Genotypes	DTF (days)					DTM (days)					LPP (no.)				
	E1	E2	E3	E4	E5	E1	E2	E3	E4	E5	E1	E2	E3	E4	E5
**G1**	45.00	40.50	45.00	47.50	42.50	85.00	85.50	83.35	83.50	88.50	45.50	40.00	42.30	36.90	43.90
**G2**	60.50	61.00	62.00	66.50	60.50	99.50	107.00	101.30	97.50	106.00	33.20	28.20	30.50	30.80	31.70
**G3**	48.50	44.00	48.50	48.00	42.00	83.00	87.00	84.80	81.00	87.50	29.60	21.30	25.30	33.20	23.70
**G4**	43.00	41.00	44.00	44.50	37.50	79.50	85.00	80.85	90.50	81.50	34.00	31.90	30.80	26.30	33.60
**G5**	50.00	45.50	49.00	52.50	47.00	79.60	81.00	77.95	83.00	82.00	33.20	29.80	31.00	27.70	32.80
**G6**	54.00	54.00	53.00	55.50	54.50	89.20	92.50	90.00	88.50	92.50	31.80	26.00	26.80	22.00	29.90
**G7**	46.50	44.00	45.00	52.50	45.50	84.50	85.00	84.45	90.00	87.50	24.50	22.10	22.10	27.60	24.10
**G8**	56.50	53.50	56.50	56.00	54.50	82.00	86.50	83.65	91.00	87.50	22.50	26.00	19.80	18.50	28.50
**G9**	51.00	50.00	50.50	49.50	49.50	83.50	85.00	82.95	85.50	86.00	23.60	19.50	19.90	27.50	21.20
**G10**	56.50	53.00	55.50	48.00	53.00	85.00	90.00	87.50	81.50	91.00	28.90	24.90	25.00	28.40	27.50
**G11**	47.00	42.50	46.00	51.50	44.50	84.50	84.00	83.95	84.50	87.50	34.80	31.60	31.00	24.90	33.60
**G12**	56.50	52.50	56.50	55.00	52.50	85.50	91.00	88.40	91.00	92.00	30.60	27.20	27.00	30.00	29.30
**G13**	56.00	53.50	56.00	55.00	53.00	91.00	93.50	91.10	91.00	93.50	36.40	26.60	34.40	26.50	27.60
**G14**	55.00	53.50	53.50	52.50	51.50	89.50	95.50	91.30	91.50	95.00	34.40	29.40	29.60	28.90	31.40
**G15**	51.00	54.00	51.00	58.00	52.50	89.50	94.50	91.00	93.00	94.00	36.10	31.50	32.80	33.60	34.00
**G16**	47.50	47.00	47.50	55.50	48.00	89.50	90.50	89.25	93.50	92.50	34.60	29.40	30.30	32.40	31.40
**G17**	49.00	44.50	48.00	48.00	46.00	86.50	86.50	84.20	84.50	87.50	25.70	19.60	18.80	19.00	22.40
**G18**	44.50	41.50	44.00	47.50	43.00	89.00	91.00	89.65	90.50	93.00	22.70	25.10	20.90	20.50	26.80
**G19**	55.50	52.50	57.00	57.50	54.50	94.50	101.00	96.75	94.00	100.00	28.00	23.60	24.70	30.00	27.70
**G20**	48.00	46.50	47.50	50.00	46.00	83.00	89.00	85.40	90.50	89.00	23.50	18.70	17.90	18.10	20.00
**G21**	50.00	44.50	49.50	48.50	45.50	80.00	84.00	82.40	88.00	86.00	26.50	24.30	24.80	24.30	26.00
**G22**	49.50	45.00	49.50	49.50	46.50	84.00	83.50	84.50	90.00	87.00	33.00	26.40	29.20	31.90	30.50
**G23**	47.00	43.50	46.00	45.50	44.00	82.00	83.50	81.45	89.50	84.00	36.80	34.40	34.60	28.80	37.10
**G24**	50.00	50.50	49.50	56.50	52.00	95.50	93.00	92.20	96.00	95.00	23.20	25.50	19.70	20.10	24.60
**Mean**	**50.75**	**48.25**	**50.44**	**52.13**	**48.58**	**86.45**	**89.38**	**87.01**	**89.15**	**90.25**	**30.55**	**26.79**	**27.05**	**27.00**	**29.14**
**S.E. (m)**	**0.72**	**0.70**	**0.86**	**0.83**	**0.83**	**1.35**	**1.35**	**1.26**	**1.34**	**1.27**	**0.50**	**0.39**	**0.41**	**0.35**	**0.50**
**C.V.**	**2.45**	**2.51**	**2.94**	**2.76**	**2.95**	**2.70**	**2.62**	**2.50**	**2.61**	**2.44**	**2.83**	**2.50**	**2.60**	**2.22**	**2.96**

DTF: Days to 50% flowering; DTM: Days to 80% maturity; LPP: Leaves per plant

Supplementary table 2.2: Comparative pooled mean performance observed in 24 Tartary buckwheat genotypes for PBP, PH and 100-SW during 2021-23.

Genotypes	PBP (no.)					PH (cm)					100-SW (g)				
	**E1**	**E2**	**E3**	**E4**	**E5**	**E1**	**E2**	**E3**	**E4**	**E5**	**E1**	**E2**	**E3**	**E4**	**E5**
**G1**	2.20	2.00	2.40	2.80	2.00	121.55	105.95	112.35	100.50	111.15	2.32	2.10	2.28	1.82	2.22
**G2**	3.00	2.40	2.80	2.60	2.00	110.65	95.85	99.69	95.02	101.39	2.59	2.16	2.46	2.48	2.25
**G3**	2.80	2.20	2.60	2.00	2.20	97.75	84.20	88.35	77.25	87.70	2.38	2.05	2.39	2.49	2.14
**G4**	3.00	2.60	3.00	3.20	2.40	99.05	87.70	89.95	84.55	91.20	2.00	1.67	1.94	1.94	1.71
**G5**	3.40	3.00	3.80	3.60	3.60	88.90	74.40	76.63	80.35	80.00	2.57	1.92	2.46	2.19	2.01
**G6**	3.40	2.80	3.20	2.20	3.20	96.45	82.96	85.83	87.75	86.46	2.17	1.82	2.08	2.01	1.89
**G7**	3.00	2.80	2.80	3.40	3.00	89.70	79.55	81.73	78.60	83.05	1.84	1.74	1.94	1.80	1.79
**G8**	2.20	2.00	2.20	2.40	2.00	97.30	84.73	89.48	78.10	88.23	2.37	2.02	2.31	2.40	2.08
**G9**	2.60	2.20	2.40	2.80	2.40	87.65	72.76	76.97	75.44	76.26	2.36	2.13	2.39	2.38	2.20
**G10**	2.60	2.00	2.80	2.60	2.40	89.85	76.65	79.38	75.25	80.15	2.30	1.89	2.28	2.05	1.94
**G11**	3.40	3.00	3.40	3.40	3.00	99.55	90.67	91.92	83.15	94.17	2.44	2.36	2.47	2.55	2.40
**G12**	3.00	2.60	2.40	3.00	2.60	100.35	86.88	90.28	78.35	90.38	2.52	2.35	2.48	2.72	2.39
**G13**	3.20	2.60	3.40	3.20	3.00	104.25	92.24	94.65	85.35	95.74	2.10	1.92	2.16	2.25	1.98
**G14**	2.80	2.00	2.60	2.60	2.60	95.70	80.99	85.82	82.80	84.49	1.89	1.57	1.75	1.81	1.60
**G15**	3.20	2.80	3.40	3.20	3.60	92.90	81.12	85.47	78.61	84.62	2.49	2.34	2.39	2.72	2.36
**G16**	3.00	2.40	2.80	2.60	2.60	102.85	88.69	95.06	82.40	92.19	2.39	2.15	2.39	2.45	2.20
**G17**	2.60	2.20	2.60	3.20	2.40	92.70	81.16	83.61	82.75	84.66	2.47	2.30	2.40	2.61	2.35
**G18**	3.40	2.60	3.00	2.40	2.80	88.00	70.70	73.20	65.75	74.20	2.15	1.95	2.07	2.13	2.00
**G19**	3.40	3.00	3.60	3.60	3.40	90.15	78.74	81.96	85.40	82.24	2.48	2.30	2.45	2.56	2.34
**G20**	2.60	2.20	2.60	2.80	2.60	90.10	73.81	78.22	70.70	77.31	2.56	2.16	2.35	2.38	2.16
**G21**	2.60	2.80	3.20	2.80	3.00	93.30	77.72	83.25	73.65	81.22	2.00	1.81	1.99	1.92	1.84
**G22**	3.00	2.60	3.20	2.20	3.00	99.95	85.72	89.93	79.75	89.22	2.31	1.89	2.09	2.06	1.96
**G23**	4.00	3.60	4.00	3.00	3.80	97.65	94.71	91.62	90.00	98.21	2.59	2.34	2.47	2.56	2.38
**G24**	2.40	2.20	2.20	2.80	2.40	82.70	72.00	74.31	65.50	75.50	2.42	2.21	2.42	2.92	2.30
**Mean**	**2.95**	**2.53**	**2.93**	**2.85**	**2.75**	**96.21**	**83.33**	**86.65**	**80.71**	**87.07**	**2.32**	**2.05**	**2.27**	**2.30**	**2.10**
**S.E. (m)**	**0.07**	**0.07**	**0.06**	**0.07**	**0.07**	**1.41**	**1.12**	**1.36**	**1.36**	**1.31**	**0.03**	**0.03**	**0.03**	**0.03**	**0.03**
**C.V.**	**3.71**	**4.44**	**3.59**	**4.00**	**4.01**	**2.54**	**2.33**	**2.72**	**2.72**	**2.61**	**2.27**	**2.61**	**2.29**	**2.37**	**2.88**

PBP: Primary branches per plant; PH: Plant height; 100-SW: 100 seed weight

Supplementary table 2.3: Comparative pooled mean performance observed in 24 Tartary buckwheat genotypes for NSPP, SPP and SYPP during 2021-23.

**Genotypes**	**NSPP (no.)**					**SPP (g)**					**SYPP (g)**				
	**E1**	**E2**	**E3**	**E4**	**E5**	**E1**	**E2**	**E3**	**E4**	**E5**	**E1**	**E2**	**E3**	**E4**	**E5**
**G1**	215.50	188.70	193.85	184.50	190.33	10.44	9.31	10.47	8.93	9.27	6.21	4.82	5.36	4.86	5.01
**G2**	168.25	159.10	163.80	145.60	162.25	9.54	7.99	9.45	7.26	7.65	4.33	3.37	3.90	3.30	3.55
**G3**	191.70	185.60	188.70	178.00	179.40	9.59	7.09	9.13	7.10	7.09	5.19	3.75	4.25	3.78	3.61
**G4**	126.30	115.80	115.15	144.00	112.20	8.16	8.20	8.02	6.55	8.30	3.30	2.08	2.86	2.03	2.28
**G5**	124.00	111.00	115.05	105.40	106.50	8.39	7.79	8.46	7.17	7.36	2.62	2.17	2.24	1.60	2.32
**G6**	104.55	90.10	92.60	115.00	91.00	7.41	7.22	7.48	7.38	7.53	2.23	1.52	1.42	1.73	1.32
**G7**	123.00	104.25	113.85	105.30	104.85	7.86	6.61	7.44	6.44	6.54	2.39	2.33	2.09	2.13	2.19
**G8**	145.00	134.00	138.55	127.00	138.33	9.62	7.74	9.64	7.76	7.89	2.84	3.13	2.46	2.16	2.89
**G9**	126.25	115.20	117.40	109.90	108.00	9.00	7.39	9.04	7.23	7.34	2.73	2.25	2.38	2.21	2.17
**G10**	137.35	134.00	137.50	132.10	133.00	7.15	6.96	7.29	6.82	6.89	4.00	3.50	3.80	3.18	3.35
**G11**	138.80	141.80	140.45	136.30	143.25	7.58	7.87	8.21	7.19	7.91	4.06	3.45	3.79	3.24	3.39
**G12**	103.50	132.25	105.40	96.90	138.20	8.43	7.48	8.49	7.41	7.69	2.62	3.17	2.27	2.33	3.12
**G13**	98.70	94.90	97.30	112.30	92.90	7.02	7.50	7.35	7.26	7.60	4.28	3.65	4.02	3.47	3.62
**G14**	100.05	111.30	100.10	92.60	115.50	8.19	7.09	8.19	6.57	6.99	2.16	1.44	1.57	1.32	1.42
**G15**	97.80	93.20	96.20	110.00	95.50	8.30	7.76	9.60	7.50	7.80	2.45	1.97	1.81	1.96	2.01
**G16**	109.15	105.40	108.65	102.40	103.10	9.05	7.05	9.09	6.81	7.14	3.19	2.47	2.75	2.41	2.50
**G17**	118.50	86.30	119.25	109.20	82.80	6.67	7.37	6.65	6.83	7.25	3.16	2.60	2.90	2.69	2.77
**G18**	137.80	126.60	139.45	133.00	117.80	8.28	7.25	8.40	7.98	7.30	4.09	3.08	3.47	3.06	3.12
**G19**	127.85	117.80	130.85	123.80	123.50	7.62	6.84	7.51	7.12	6.56	3.59	3.08	3.52	3.08	3.17
**G20**	138.45	133.00	136.60	130.10	137.50	8.62	6.84	7.74	6.49	6.84	4.00	3.49	3.47	3.32	3.42
**G21**	122.45	157.60	120.70	114.60	163.00	8.78	6.90	8.66	6.63	6.91	2.62	2.49	2.32	2.05	2.45
**G22**	97.95	85.70	98.20	91.80	83.60	8.77	8.06	9.60	7.53	7.90	2.45	2.12	1.95	1.72	1.95
**G23**	97.15	83.90	96.45	91.70	85.50	9.16	8.38	9.03	8.35	8.63	2.68	2.09	2.30	2.09	2.20
**G24**	93.85	91.50	97.40	92.50	93.00	7.50	6.87	7.25	6.66	6.95	2.48	2.16	2.08	2.12	2.18
**Mean**	**126.83**	**120.79**	**123.48**	**120.17**	**120.88**	**8.38**	**7.48**	**8.42**	**7.21**	**7.47**	**3.32**	**2.76**	**2.87**	**2.57**	**2.75**
**S.E. (m)**	**1.84**	**1.92**	**1.74**	**1.67**	**2.04**	**0.10**	**0.11**	**0.14**	**0.12**	**0.12**	**0.06**	**0.05**	**0.05**	**0.05**	**0.05**
**C.V.**	**2.51**	**2.76**	**2.44**	**2.41**	**2.92**	**2.14**	**2.56**	**2.88**	**2.83**	**2.69**	**2.86**	**3.21**	**3.10**	**3.53**	**3.27**

NSPP: Number of seed per plant; SPP: Straw yield per plant; SYPP: Seed yield per plant

Supplementary table 2.4: Comparative pooled mean performance observed in 24 Tartary buckwheat genotypes for Ca, P and Mg during 2021-23.

**Genotypes**	**Ca (mg per 100 g)**					**P (mg per 100 g)**					**Mg (mg per 100 g)**				
	**E1**	**E2**	**E3**	**E4**	**E5**	**E1**	**E2**	**E3**	**E4**	**E5**	**E1**	**E2**	**E3**	**E4**	**E5**
**G1**	57.70	51.70	59.30	56.70	56.40	362.20	358.10	368.80	378.70	391.40	216.30	222.00	234.50	221.80	227.50
**G2**	55.50	51.10	56.70	56.10	54.20	364.20	353.50	343.80	330.70	314.40	229.50	225.10	239.60	224.90	209.70
**G3**	48.60	49.60	44.40	54.60	52.30	364.60	354.90	370.20	355.10	343.80	204.20	193.70	178.20	193.50	192.30
**G4**	56.10	60.10	61.50	65.10	54.80	349.40	359.70	340.00	354.90	366.60	204.30	217.00	205.50	216.80	219.60
**G5**	59.30	53.30	57.70	58.30	48.00	375.90	386.20	356.50	367.40	359.10	199.30	213.80	203.30	213.60	193.40
**G6**	56.80	65.20	60.40	64.30	55.50	361.90	383.20	397.50	407.40	413.10	195.90	183.40	192.90	183.20	161.00
**G7**	51.50	47.50	50.70	52.50	50.20	354.70	345.00	332.30	322.20	331.90	227.60	224.20	233.70	224.00	231.80
**G8**	53.60	59.60	57.20	64.60	52.30	365.80	381.10	397.40	387.30	374.00	230.80	222.10	242.60	221.90	225.70
**G9**	57.80	49.80	59.60	54.80	56.50	349.50	329.80	341.10	320.00	304.70	198.60	197.10	186.60	196.90	199.70
**G10**	51.70	39.70	44.90	44.70	50.40	372.50	342.80	331.10	341.00	355.70	205.30	203.90	208.60	203.70	186.50
**G11**	60.50	58.20	59.30	63.20	51.30	360.30	374.60	382.90	367.80	378.50	205.70	200.20	199.60	200.00	182.80
**G12**	46.20	51.60	45.60	56.60	55.50	369.50	356.80	351.10	371.00	380.70	210.40	200.90	190.00	200.70	172.50
**G13**	53.90	60.30	55.90	65.30	52.60	354.70	371.00	361.30	391.20	380.90	216.30	229.80	223.30	229.60	232.40
**G14**	57.00	61.40	62.80	66.40	55.70	346.30	357.60	370.90	376.80	366.50	205.70	221.20	210.70	221.00	198.80
**G15**	50.40	45.60	52.00	50.60	49.10	359.90	348.20	363.50	373.40	352.10	194.50	180.00	192.50	179.80	192.60
**G16**	56.80	53.10	58.70	58.10	48.60	355.20	363.50	348.80	335.70	345.40	204.70	194.20	208.70	194.00	216.80
**G17**	49.60	51.90	46.70	56.90	53.00	367.10	361.40	350.70	335.60	320.30	209.50	200.00	184.50	199.80	198.60
**G18**	52.60	48.60	55.00	53.60	51.30	365.30	355.60	373.90	388.80	408.50	212.40	222.90	211.40	222.70	225.50
**G19**	62.80	57.60	62.00	62.60	51.10	354.60	367.90	384.20	395.10	424.80	208.50	219.00	208.50	218.80	198.60
**G20**	49.80	55.80	51.00	60.80	48.50	347.20	362.50	332.80	342.70	348.40	127.60	219.40	228.90	219.20	197.00
**G21**	55.40	49.40	52.60	54.40	54.10	346.10	331.40	345.70	335.60	345.30	208.80	199.30	208.80	199.10	206.90
**G22**	53.20	48.80	46.40	53.80	51.90	369.20	358.50	345.80	335.70	322.40	214.70	230.20	250.70	230.00	233.80
**G23**	49.50	56.70	52.70	61.70	48.20	360.40	378.70	395.00	373.90	358.60	211.80	192.30	181.80	192.10	194.90
**G24**	51.40	57.80	63.00	62.80	50.10	357.30	373.60	384.90	394.80	409.50	205.70	176.20	190.70	176.00	158.80
**Mean**	**54.07**	**53.52**	**54.84**	**58.27**	**52.15**	**359.74**	**360.65**	**361.26**	**361.78**	**362.36**	**206.17**	**207.83**	**208.98**	**207.63**	**202.38**
**S.E. (m)**	**0.86**	**0.75**	**0.87**	**0.89**	**0.74**	**5.65**	**6.50**	**5.42**	**5.32**	**5.71**	**3.17**	**2.67**	**2.72**	**3.15**	**2.96**
**C.V.**	**2.76**	**2.43**	**2.75**	**2.64**	**2.46**	**2.72**	**3.12**	**2.60**	**2.55**	**2.73**	**2.67**	**2.23**	**2.25**	**2.06**	**2.53**

Ca: Calcium; P: Phosphorus; Mg: Magnesium

Supplementary table 2.5: Comparative pooled mean performance observed in 24 Tartary buckwheat genotypes for Fe, Zn and PC during 2021-23.

Genotypes	Fe (mg per 100 g)					Zn (mg per 100 g)					PC (g per 100 g)				
	**E1**	**E2**	**E3**	**E4**	**E5**	**E1**	**E2**	**E3**	**E4**	**E5**	**E1**	**E2**	**E3**	**E4**	**E5**
**G1**	4.20	4.30	4.20	4.40	4.50	3.10	3.20	3.40	3.40	3.30	11.50	10.30	11.62	10.06	10.86
**G2**	4.20	4.40	4.60	4.90	5.10	3.20	3.40	3.30	3.60	3.20	13.80	12.92	13.96	13.40	12.20
**G3**	4.10	4.00	3.80	3.60	3.50	3.00	2.90	3.10	3.10	3.10	11.60	10.80	11.72	9.36	10.96
**G4**	4.00	4.20	3.90	4.00	4.20	2.60	2.80	2.60	3.00	3.10	12.43	13.23	13.10	14.75	15.35
**G5**	3.80	3.60	3.80	3.70	3.50	2.90	2.70	2.90	2.90	2.60	13.67	14.47	14.12	16.47	15.35
**G6**	4.10	3.80	3.70	3.60	3.30	2.60	2.30	2.40	2.50	2.20	12.89	14.57	13.35	12.33	13.37
**G7**	3.60	3.80	3.90	3.70	3.90	3.10	3.30	3.40	3.50	3.10	12.56	11.76	12.98	9.68	8.40
**G8**	3.90	3.80	3.90	4.00	3.90	2.70	2.60	2.60	2.80	2.70	13.87	15.07	14.23	17.71	16.83
**G9**	3.60	3.70	3.90	3.70	3.80	2.90	3.00	2.90	3.20	2.80	14.12	12.52	13.98	14.68	15.64
**G10**	3.70	3.80	3.70	3.90	4.00	3.00	2.80	3.00	3.00	3.00	12.75	10.35	13.12	10.45	10.91
**G11**	3.50	3.70	3.90	4.20	4.40	2.60	2.80	2.60	3.00	3.10	13.88	15.00	13.96	12.84	12.04
**G12**	4.20	4.10	3.90	3.70	3.60	2.90	2.80	2.50	3.00	2.60	12.22	11.18	11.98	11.58	9.90
**G13**	4.20	4.40	4.10	4.20	4.40	3.10	3.30	3.50	3.50	3.40	14.38	15.66	13.87	17.34	18.14
**G14**	3.90	3.70	3.80	3.70	3.50	3.10	2.90	2.80	3.10	2.80	13.60	14.48	13.42	16.08	14.88
**G15**	3.70	3.40	3.50	3.40	3.10	2.80	2.50	2.60	2.70	2.40	12.76	11.80	12.60	12.68	14.28
**G16**	3.80	4.00	4.20	4.00	4.20	2.70	2.90	3.00	3.10	2.70	12.40	13.04	12.36	11.44	13.84
**G17**	4.10	4.00	3.90	4.00	3.90	2.80	2.70	2.90	2.90	2.80	12.20	11.72	12.23	11.24	10.12
**G18**	4.20	4.30	4.50	4.30	4.40	2.60	2.70	2.60	2.90	2.50	13.12	12.32	12.86	11.52	12.56
**G19**	4.30	4.40	4.20	4.40	4.50	3.10	3.20	3.40	3.40	3.40	13.13	14.17	13.19	10.57	9.29
**G20**	3.70	3.90	3.60	3.90	4.10	2.60	2.80	2.60	3.00	3.10	13.21	14.41	13.29	17.13	16.25
**G21**	3.90	3.80	4.00	3.90	3.80	3.30	3.10	3.30	3.30	3.10	12.55	11.35	12.48	12.71	13.67
**G22**	3.70	3.90	3.80	3.70	3.90	2.90	3.10	3.10	3.30	3.10	12.11	11.23	12.19	11.39	10.75
**G23**	4.10	3.90	4.20	3.80	3.60	3.10	2.90	2.90	3.10	2.90	13.01	14.45	13.14	16.37	16.85
**G24**	4.00	3.70	4.20	3.90	3.60	2.80	2.50	2.70	2.70	2.50	12.22	13.50	12.13	11.26	12.06
**Mean**	**3.94**	**3.94**	**3.97**	**3.94**	**3.95**	**2.90**	**2.88**	**2.92**	**3.08**	**2.90**	**12.92**	**12.93**	**12.99**	**13.04**	**13.10**
**S.E. (m)**	**0.05**	**0.05**	**0.06**	**0.06**	**0.06**	**0.04**	**0.05**	**0.05**	**0.04**	**0.04**	**0.19**	**0.17**	**0.20**	**0.20**	**0.19**
**C.V.**	**2.26**	**2.24**	**2.59**	**2.77**	**2.55**	**2.57**	**3.04**	**2.98**	**2.18**	**2.19**	**2.61**	**2.25**	**2.66**	**2.67**	**2.52**

Fe: Iron; Zn: Zinc; PC: Protein content

Supplementary table 3: Genotype classification in four different quadrants based on 'mean *vs* WAAS biplot'.

Trait	Quadrant I *(Below Avg, High WAAS)*	Quadrant II *(Above Avg, High WAAS)*	Quadrant III *(Below Avg, Low WAAS)*	Quadrant IV *(Above Avg, Low WAAS)*
**Agro-morphological traits**				
DTF	G16, G7, G11	G10, G15, G24, G14	G1, G3, G21, G23, G17, G5, G22, G18, G4, G20	G9, G13, G12, G2, G8, G6, G19
DTM	G4, G10, G23, G8, G3, G22	G2, G19	G7, G21, G1, G20, G17, G11, G9, G5	G6, G14, G24, G16, G13, G18, G12, G15
LPP	G3, G8, G9, G7, G18, G19, G6	G11, G23	G24, G10, G17, G20, G21	G13, G4, G22, G12, G5, G16, G2, G14, G15
PBP	G1, G3, G17	G6, G22, G23, G18	G24, G12, G8, G9, G2, G20, G10, G16, G14	G4, G7, G21, G15, G11, G13, G19, G5
PH	G19, G5	G6, G12, G23	G9, G17, G18, G21, G14, G7, G24, G20, G10, G15	G3, G16, G8, G11, G1, G22, G13, G4, G2
100-SW	G1, G10	G24, G5, G15, G12	G7, G22, G21, G6, G13, G14, G4, G18	G3, G17, G12, G20, G11, G8, G2, G19, G16, G9, G23
NSPP	G12, G17, G14, G6	G21, G4	G13, G15, G22, G7, G23, G9, G5, G24, G16	G18, G11, G19, G8, G20, G10, G2, G3, G1
SPP	G17, G13, G16, G11, G6, G21	G3, G4, G2, G8	G10, G20, G24, G19, G7, G14	G9, G22, G23, G1, G15, G18, G5, G12
SYPP	G12, G8, G4, G7	G3, G1	G21, G24, G6, G5, G22, G15, G9, G16, G17, G23, G14	G18, G2, G1, G19, G8, G20, G10, G13
**Nutritional traits**				
Ca	G10	G9, G24	G2, G3, G7, G12, G15, G17, G20, G21, G22, G23	G5, G4, G1, G6, G8, G2, G11, G14, G16, G19, G13
P	G9, G2, G22, G17	G6, G18, G24, G19	G3, G4, G7, G10, G14, G15, G20, G21	G13, G23, G14, G1, G5, G8, G11, G12
Mg	G12, G20, G24	G22	G3, G5, G6, G9, G10, G15, G17, G21	G2, G1, G4, G7, G8, G19, G13, G14, G18
Fe	G3, G6, G11, G12, G15, G23	G2	G5, G7, G8, G9, G10, G14, G20, G21, G22	G1, G4, G13, G16, G17, G18, G19
Zn	G6, G15, G12, G4, G20, G11	—	G5, G8, G16, G17, G18, G24	G1, G2, G3, G7, G9, G10, G13, G19, G21, G22, G23
PC	G7, G19	G8, G23, G20, G13	G1, G3, G10, G16, G17, G12, G22, G24, G18, G21, G15	G4, G2, G5, G6, G11, G9, G14
